# Supplementary material for: Laser interstitial thermal therapy and adjuvant pembrolizumab in recurrent high-grade astrocytoma: a Phase 1/randomized Phase 2b trial
Source: Nat Commun. 2026 Feb 26;17:1763. doi: 10.1038/s41467-026-69522-w (PMC12946167; doi:10.1038/s41467-026-69522-w)
Supplement: Supplementary file 1 — Supplementary Information [file 41467_2026_69522_MOESM1_ESM.pdf]

Supplementary Table S1 (related to **Table 1** and **Figures 1-4**): **Demographic and Clinical Characteristics for wt-*IDH* GBM versus CHC Cohorts**

CHARACTERISTICS	LITT+PEM n = 29	LITT+non-ICI n = 13	Overall n = 42	P-Value
**Age, Median (IQR)**	61 (49,67)	67 (47,74)	63 (50,68)	*0.165*
**Sex, n (%)**				*0.514*
Female	14 (48.3)	8 (61.5)	22 (52.4)	
Male	15 (51.7)	5 (38.5)	20 (47.6)	
**Initial diagnosis, n (%)**				*1.000*
Astrocytoma	0 (0.0)	0 (0.0)	0 (0.0)	
GBM	29 (100.0)	13 (100.0)	42 (0.0)	
***IDH* mutation, n (%)**				*1.000*
Wildtype	29 (100.0)	13 (100.0)	42 (0.0)	
Mutation	0 (0.0)	0 (0.0)	0 (0.0)	
***MGMT* status, n (%)**				*0.37*
Unknown	4 (13.8)	0 (0.0)	4 (9.5)	
Unmethylated	11 (37.9)	6 (46.2)	17 (40.5)	
Methylated	14 (48.3)	7 (53.8)	21 (50.0)	
**Prior recurrence, n (%)**				*0.54*
2nd Recurrence	26 (89.7)	13 (100.0)	18 (85.7)	
≥ 3rd Recurrence	3 (10.3)	0 (0.0)	3 (14.3)	
**Tumor location, n (%)**				*0.027*
Frontal lobe	11 (37.9)	8 (61.5)	19 (45.2)	
Temporal lobe	10 (34.5)	0 (0.0)	10 (23.8)	
Parietal lobe	6 (20.7)	3 (23.1)	9 (21.4)	
Occipital lobe	2 (6.9)	0 (0.0)	2 (4.8)	
Others	0 (0.0)	2 (15.4) (1 cerebellum; 1 corpus callosum)	2 (4.8)	
**Institution, n (%)**				*0.008*
FL	12 (41.4)	0 (0.0)	12 (28.6)	
WU	17 (58.6)	13 (100)	30 (71.4)	

Data are median (IQR) or n (%). P values were calculated using the Wilcoxon rank-sum test for continuous variables and Fisher's exact test for categorical variables.

Supplementary Tables S2 (related to Figures 3, 4): Multivariable Analysis

Population	Survival	Parameter	P-value	HR	95% CI	
**ITT** *	**OS**	Arm (LITT+PEM vs. NLS+PEM)	0.1585	0.267	0.043	1.674
		Age	0.6849	0.975	0.861	1.104
		MGMT (Methylated vs. Unmethylated)	0.7573	1.444	0.140	14.851
		Sex (Male vs. Female)	0.2937	0.389	0.067	2.268
		Center (WashU vs. UFlorida)	0.2213	0.261	0.030	2.247
		Recurrence Number	0.2102	0.069	0.001	4.516
		KPS	0.6586	0.972	0.858	1.101
	**PFS**	Arm LITT+PEM vs. NLS+PEM	0.2367	0.364	0.068	1.941
		Age	0.4317	0.955	0.851	1.072
		MGMT (Methylated vs. Unmethylated)	0.6122	1.907	0.157	23.138
		Sex (Male vs. Female)	0.0988	0.141	0.014	1.444
		Center (WashU vs. UFlorida)	0.1740	0.279	0.044	1.758
		Recurrence Number	0.4653	0.301	0.012	7.537
		KPS	0.3565	0.956	0.869	1.052
**Per Protocol**	**OS**	Arm (LITT+PEM vs. NLS+PEM)	0.0061	0.147	0.037	0.578
		Age	0.2752	0.971	0.922	1.023
		IDH Mutation (Mutated vs. Wildtype)	0.0665	0.188	0.031	1.121
		MGMT (Methylated vs. Unmethylated)	0.1130	0.405	0.132	1.239
		Sex (Male vs. Female)	0.3395	0.645	0.262	1.586
		Center (WashU vs. UFlorida)	0.5782	0.728	0.238	2.230
		Recurrence Number	0.0497	0.180	0.032	0.998
		KPS	0.1914	0.964	0.913	1.018
	**PFS**	Arm (LITT+PEM vs. NLS+PEM)	0.0084	0.186	0.053	0.649
		Age	0.1500	0.960	0.909	1.015
		IDH Mutation (Mutated vs. Wildtype)	0.2841	0.402	0.076	2.131
		MGMT (Methylated vs. Unmethylated)	0.5669	0.701	0.208	2.366
		Sex (Male vs. Female)	0.2496	0.542	0.191	1.538
		Center (WashU vs. UFlorida)	0.5747	0.739	0.258	2.123
		Recurrence Number	0.0588	0.188	0.033	1.064
		KPS	0.1577	0.963	0.915	1.015

- The variable *IDH* mutation status has only one level after 5 observations with missing were deleted. Therefore, *IDH* mutation status was excluded from the multivariate cox model in the ITT analysis.
- Univariate and multivariable Cox proportional-hazards models adjusted for age, sex, *IDH* mutation, *MGMT* methylation, enrolling center, baseline KPS, and number of prior recurrences provided hazard ratios (HR) with 95% confidence intervals and p-values. P values are nominal and are not adjusted for multiple comparisons. All tests were two-sided at α=0.05. Analyses were performed in SAS v9.4 (Cary, NC).

Supplementary Tables S3 (related to Figures 3, 4): Duration of Response					
**Variable**	Mean	Std Dev	Median	Minimum	Maximum
**Duration of Response (months)**	4.4	7.4	1.8	0.00	32.8

Duration of Response = Time between date of best scan result and date of progression

Supplementary Table S4 (related to **Figure 5**): **Cluster proportion fold changes in key innate immune cell types following NLS vs LITT**

**Cell type**	**Time point 1**	**Time point 2**	**Comparison**	**LogFC**	**P-value**	**Significance**
Classical Monocytes	Pre-procedure	C1	NLS+PEM vs LITT+PEM	0.35	0.68	ns
Non-classical Monocytes	Pre-procedure	C1	NLS+PEM vs LITT+PEM	-0.64	0.042	*
cDCs	Pre-procedure	C1	NLS+PEM vs LITT+PEM	-0.20	1.00	ns
pDCs	Pre-procedure	C1	NLS+PEM vs LITT+PEM	-0.55	0.58	ns
NKs	Pre-procedure	C1	NLS+PEM vs LITT+PEM	0.24	0.77	ns

P values are from two-sided paired Wilcoxon signed-rank tests comparing pre- vs post-procedure subset frequencies within individuals.

Supplementary Table S5 (related to **Figure 6**): **Cluster proportion fold changes in T cell subtypes in C1 vs C2**

Cell type	Time point 1	Time point 2	Comparison	LogFC	P-value	Significance
Activated CD4	C1	C2	NLS+PEM vs LITT+PEM	-0.55	0.32	ns
Effector CD8	C1	C2	NLS+PEM vs LITT+PEM	0.13	0.51	ns
Effector Memory Precursor CD8	C1	C2	NLS+PEM vs LITT+PEM	-0.23	0.57	ns
Exhausted T	C1	C2	NLS+PEM vs LITT+PEM	0.54	1.00	ns
Gamma/Delta T	C1	C2	NLS+PEM vs LITT+PEM	0.22	0.13	ns
Naïve CD4	C1	C2	NLS+PEM vs LITT+PEM	-1.54	0.80	ns
Memory CD4	C1	C2	NLS+PEM vs LITT+PEM	0.024	0.53	ns
Stem-like CD8	C1	C2	NLS+PEM vs LITT+PEM	-0.49	0.15	ns
Effector Memory CD8	C1	C2	NLS+PEM vs LITT+PEM	0.31	0.59	ns
Central Memory CD8	C1	C2	NLS+PEM vs LITT+PEM	0.44	0.95	ns
GZMK^{+} Effector Memory CD8	C1	C2	NLS+PEM vs LITT+PEM	-0.29	0.10	ns
Cycling Effector	C1	C2	NLS+PEM vs LITT+PEM	0.62	0.064	ns

P values are from two-sided paired Wilcoxon signed-rank tests comparing pre- vs post-procedure subset frequencies within individuals.

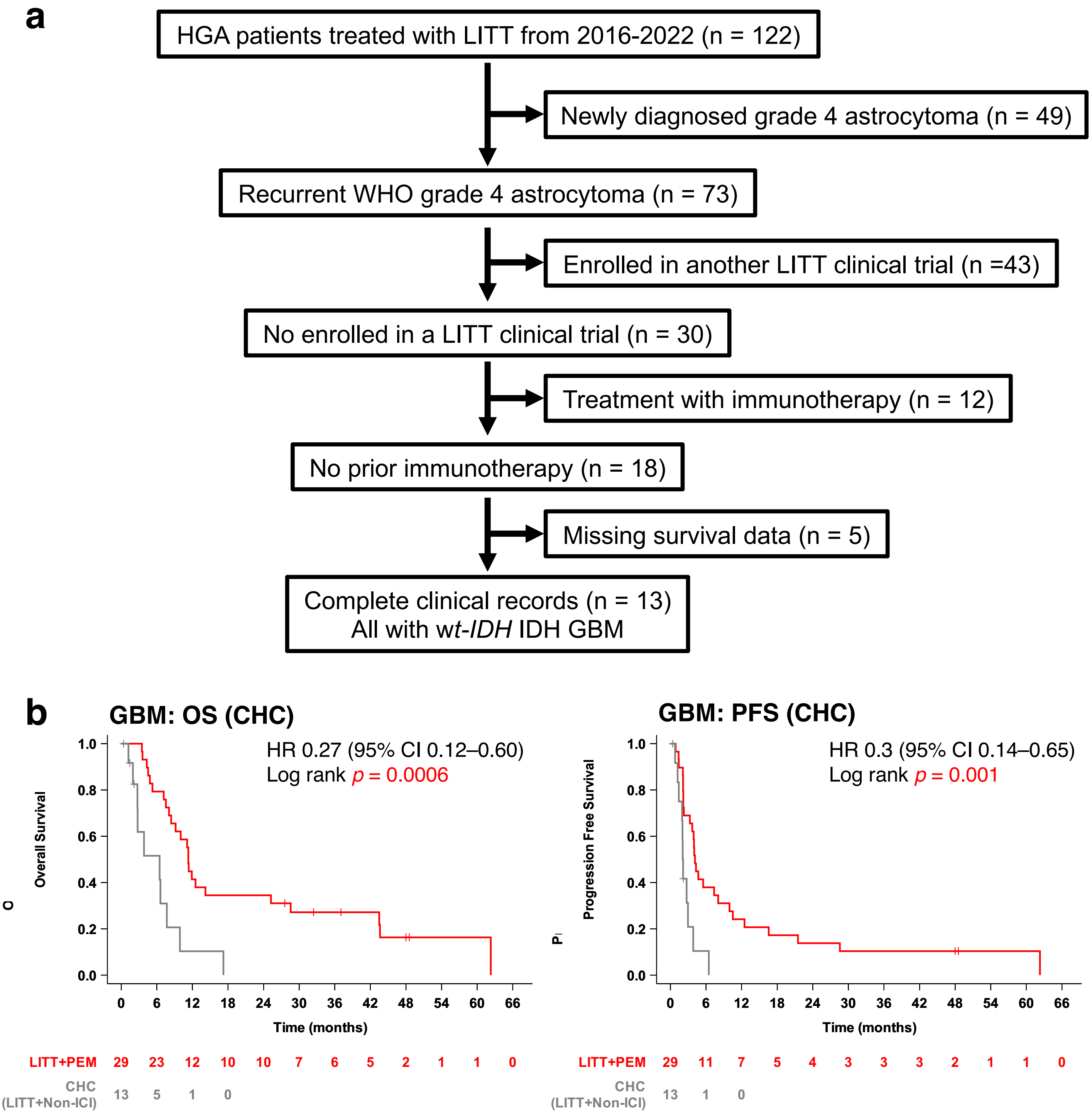

Supplementary Figure S1 (related to **Figures 3, 4**): **Survival of wt-*IDH* GBM vs CHC patients**

a) Creation of the CHC cohort. A diagram detailing the selection of patients appropriate for the CHC cohort. Patients were treated between 2017 and 2022 at Washington University. All 13 CHC patients were wt-*IDH* GBM.

b) Median OS and PFS were 11.3 months (95% CI, 8.1–25.3) and 4.1 months (95% CI, 2.3–8.1), respectively, in the LITT+PEM arm, compared to 6.5 months (95% CI, 2.0–7.7) and 2.1 months (95% CI, 1.2–3.0) in the CHC (LITT+Non-ICI) control arm. Survival was measured from the day of LITT. Kaplan-Meier estimates were used for survival analysis, with comparisons made using the log-rank test.

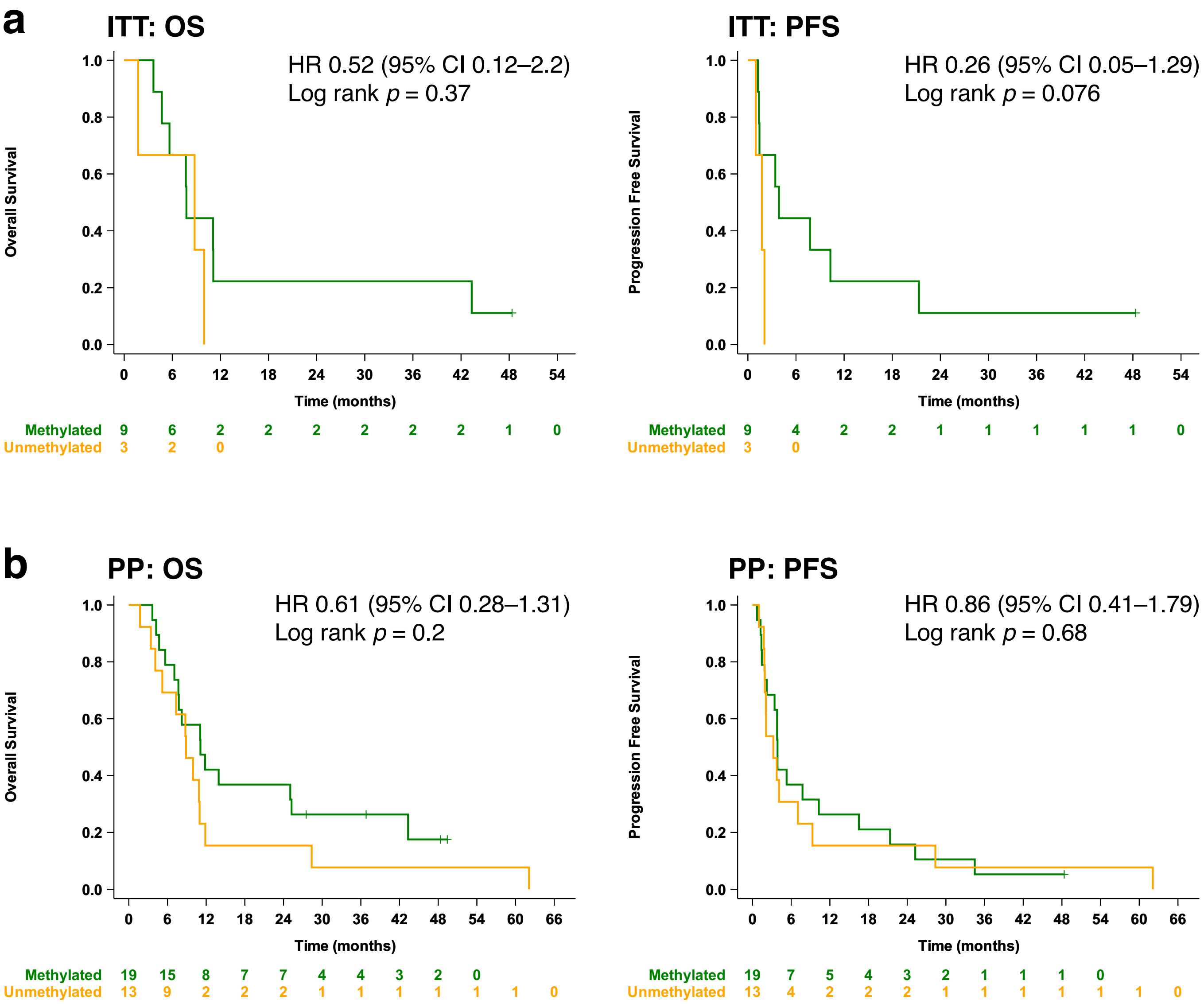

Supplementary Figure S2 (related to **Figures 3, 4**)**: Survival Analysis of the ITT and PP populations as a function of *MGMT* promoter methylation status.**

a) For the Phase 2's randomized ITT population with available *MGMT* promoter methylation status (n = 12), median OS and PFS were 7.8 months (95% CI, 3.7–43.3) and 3.9 months (95% CI, 1.3–21.3), respectively, for methylated patients, compared to 8.8 months (95% CI, 1.7–N/A) and 1.7 months (95% CI, 1.0–N/A) for unmethylated patients.

b) For the PP population with available *MGMT* promoter methylation status (n = 32), median OS and PFS were 11.1 months (95% CI, 8.0–25.3) and 3.9 months (95% CI, 1.9–10.3), respectively, for methylated patients, compared to 8.9 months (95% CI, 4.1–11.0) and 3.2 months (95% CI, 1.8–7.0) for unmethylated patients.

Survival was measured from cycle 1, day 1 of pembrolizumab administration. Kaplan-Meier estimates were used for survival analysis, with comparisons made using the log-rank test.

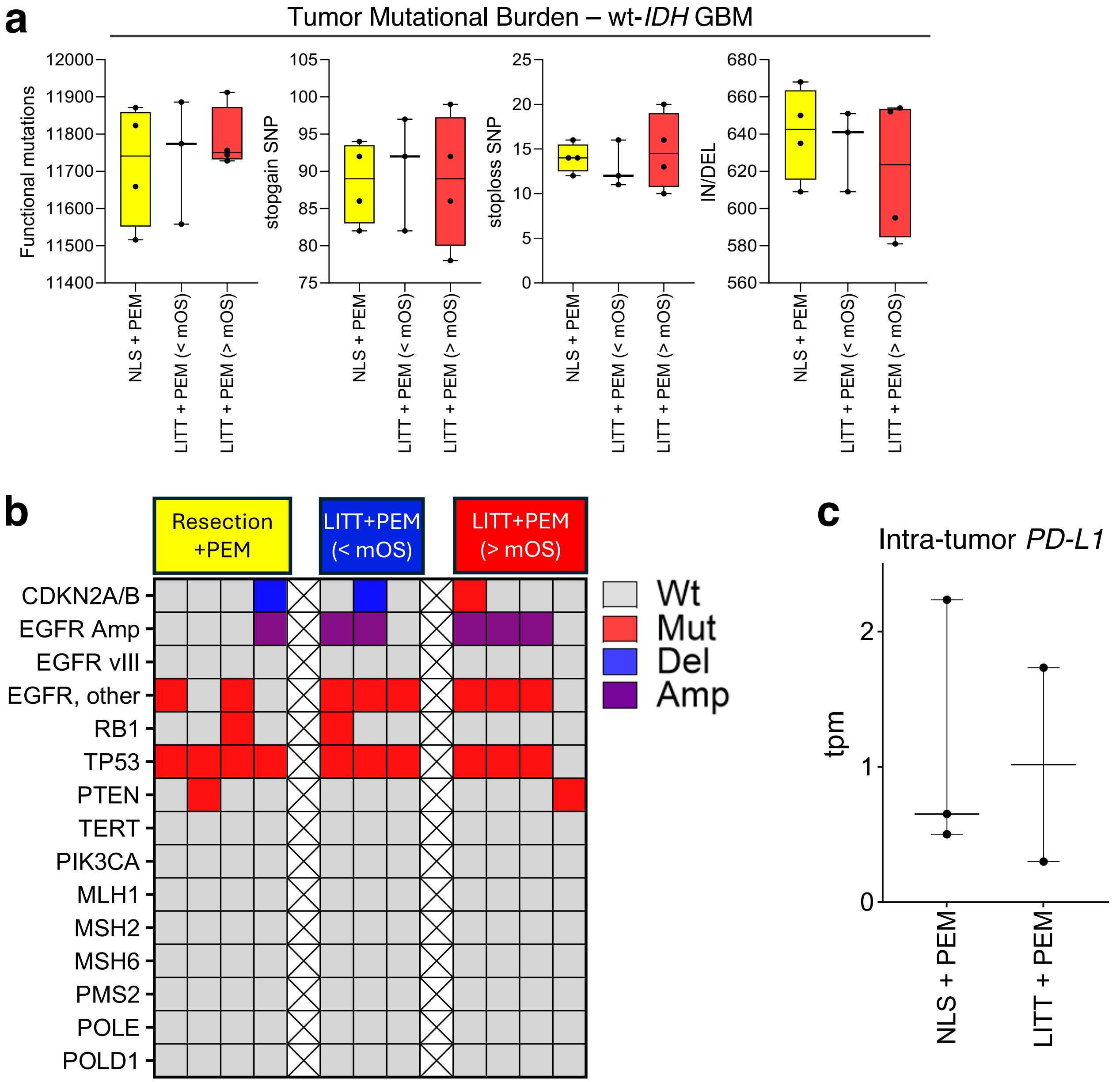

Supplementary Figure S3 (related to **Figures 3, 4**): **No large-scale genomic differences in recurrent tumor samples from NLS+PEM and LITT+PEM patients.**

a) Combination box-and-whisker and dot plots of total functional mutational burden, stop-gain and stop-loss SNP, and insertions/deletions (IN/DEL), showing no significant differences in tumor mutational burden between these patient groups. N = 4, NLS+PEM; n = 3, LITT+PEM (< mOS); n = 4, LITT+PEM (> mOS). Data are represented as mean ± SEM. The whiskers are the minimum and maximum values, the lower and upper box edges the 25th and 75th percentage values, respectively, and the lines within the boxes the median. Comparisons were performed using unpaired Student's T-test with a 2-tailed distribution.

b) Mutations (Mut) and copy number variation (deletion [Del] or amplification [Amp]) profiles of genes with known prognostic significance in high-grade glioma and immune-oncology in the same recurrent tumor samples. N = 4, NLS+PEM; n = 3, LITT+PEM (< mOS); n = 4, LITT+PEM (> mOS).

c) PD-L1 expression (transcripts per million, tmp) in bulk RNA-seq of available tumor tissue. N = 3, NLS+PEM; n = 2, LITT+PEM.

Source data are provided as a Source Data file.

A Phase I/II Study Testing the Safety, Toxicities, and Efficacy of MK-3475 in Combination with MRI-guided Laser Ablation in Recurrent Malignant Gliomas

Washington University School of Medicine, Division of Oncology
660 South Euclid Avenue, Campus Box 8056, St. Louis, MO 63110

Protocol #: 201501072
Version Date: 10/06/2021

**Coordinating Center:**	Washington University School of Medicine
**PI and IND Holder:**	Milan G. Chheda, M.D.<br>**Phone:** (314) 747-2712<br>**E-mail:** mchheda@wustl.edu
**University of Florida PI:**	David D. Tran, M.D., Ph.D.<br>**E-mail:** david.tran@neurosurgery.ufl.edu

**Sub-Investigators**	**Institution**	**Modality**
Tanner M. Johanns, M.D., Ph.D.	Washington University	Medical Oncology
Albert Kim, M.D., Ph.D.	Washington University	Neurosurgery
Eric Leuthardt, M.D.	Washington University	Neurosurgery
Esther Lu, Ph.D.	Washington University	Biostatistics
Robert D. Schreiber, Ph.D.	Washington University	Pathology & Immunology
Maryam Rahman, M.D.	University of Florida	Neurosurgery
Duane Mitchell, M.D., Ph.D.	University of Florida	Neurosurgery
Gregory Murad, M.D.	University of Florida	Neurosurgery
Ashley Ghiaseddin, M.D.	University of Florida	Neuro-Oncology
Steven Roper, M.D.	University of Florida	Neurosurgery

**Study Drug:**	MK-3475 (pembrolizumab, Keytruda)
**IND #:**	125099
**Clinical Trials.gov #:**	NCT02311582

CONFIDENTIAL
The information contained in this document is regarded as confidential and, except to the extent necessary to obtain informed consent, may not be disclosed to another party unless law or regulations require such disclosure. Persons to whom the information is disclosed must be informed that the information is confidential and may not be further disclosed by them

A Phase I/Phase II Study Testing the Safety, Toxicities, and Efficacy of MK-3475 in Combination with MRI-guided Laser Ablation in Recurrent Malignant Gliomas

Protocol Revision History

**Initial Approval Version**	**01/16/2015**
**Amendment #1 Version**	**04/16/2015**
**Amendment #2 Version**	**09/16/2015**
**Amendment #3 Version (PRMC approved only)**	**02/05/2016**
**Amendment #3 Version (HRPO requested changes)**	**03/25/2016**
**Amendment #4 Version**	**06/15/2016**
**Amendment #5 Version**	**09/22/2016**
**Amendment #6 Version**	**01/12/2017**
**Amendment #7 Version**	**09/20/2017**
**Amendment #8 Version**	**10/19/2017**
**Amendment #9 Version**	**12/05/2017**
**Amendment #10 Version**	**01/18/2017**
**Amendment #11 Version**	**02/21/2018**
**Amendment #12 Version**	**06/19/2019**
**Amendment #13 Version**	**01/06/2021**
**Amendment #14 Version**	**05/27/2021**
**Amendment #15 Version**	**08/03/2021**
**Amendment #16 Version**	**10/06/2021**

A Phase I/II Study Testing the Safety, Toxicities, and Efficacy of MK-3475 in Combination with MRI-guided Laser Ablation in Recurrent Malignant Gliomas

Principal Investigator Signature Page

Principal Investigator:	Milan G. Chheda, M.D.
	________________________ __________<br>Signature of Investigator Date
	________________________<br>Printed Name of Investigator
	By my signature, I agree to personally supervise the conduct of this study and to ensure its conduct in compliance with the protocol, informed consent, IRB/HRPO procedures, the Declaration of Helsinki, ICH Good Clinical Practices guidelines, and the applicable parts of the United States Code of Federal Regulations or local regulations governing the conduct of clinical studies.

A Phase I/II Study Testing the Safety, Toxicities, and Efficacy of MK-3475 in Combination with MRI-guided Laser Ablation in Recurrent Malignant Gliomas

SCHEMA

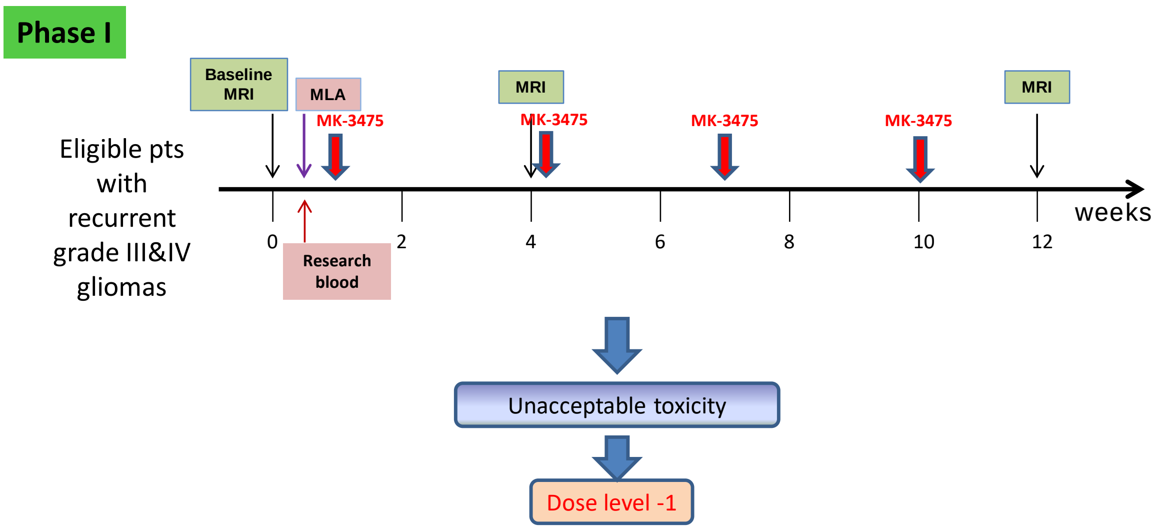

Phase I sample size: maximal 18
Phase II sample size: 32 evaluable patients with up to 39 enrollments

Dose Escalation Schedule (Phase I portion)	
**Dose Level**	**MK-3475 Dose**
Level 1 (Starting Dose)	100 mg IV q3wks
Level 2	150 mg IV q3wks
Level 3	200 mg IV q3wks

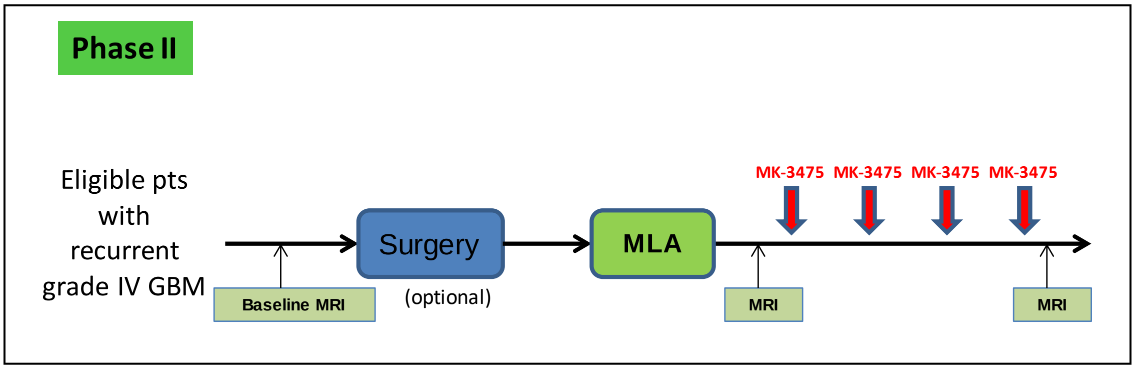

As of Amendment 12, patients will no longer be randomized in the phase II portion of this trial. Phase II patients enrolled after the approval of Amendment 12 will participate in a single treatment arm with MK-3475 and MLA and follow the schedule of events in Section 10.2.1 of the protocol.

Glossary of Abbreviations

AE	Adverse event
ALT (SGPT)	Alanine transaminase (serum glutamate pyruvic transaminase)
ANC	Absolute neutrophil count
AST (SGOT)	Aspartate transaminase (serum glutamic oxaloacetic transaminase)
BBB	Blood brain barrier
B-HCG	Beta human chorionic gonadotropin
CBC	Complete blood count
CFR	Code of Federal Regulations
CNS	Central nervous system
CRF	Case report form
CT	Computed tomography
CTCAE	Common Terminology Criteria for Adverse Events
CTEP	Cancer Therapy Evaluation Program
DLTs	Dose Limiting Toxicities
DSM	Data and Safety Monitoring
ECI	Event of clinical interest
FDA	Food and Drug Administration
FNA	Fine needle aspiration
FWA	Federal wide assurance
GBM	Glioblastoma multiforme
HIV	Human Immunodeficiency Virus
HRPO	Human Research Protection Office (IRB)
IB	Investigator's brochure
IHC	Immunohistochemistry
IND	Investigational New Drug
INR	International normalized ratio
irAE	Immune-related adverse event
IRB	Institutional Review Board
IULN	Institutional upper limit of normal
IV	Intravenous (i.v.)
MLA	MRI-guided laser ablation
MRI	Magnetic resonance imaging
MTD	Maximum tolerated dose
NCI	National Cancer Institute
NIH	National Institutes of Health
NSCLC	Non-small cell lung cancer
OHRP	Office of Human Research Protections
OS	Overall survival
PFS	Progression-free survival
PI	Principal investigator

PK	Pharmacokinetic
PT	Prothrombin time
PTT	Partial thromboplastic time
QASMC	Quality Assurance and Safety Monitoring Committee
RANO	Response Assessment in Neuro-Oncology
SAE	Serious adverse event
SCC	Siteman Cancer Center
SUSAR	Suspected unexpected serious adverse reaction
TIL	Tumor-infiltrating lymphocytes
TSH	Thyroid stimulating hormone
UPN	Unique patient number

Table of Contents

SCHEMA 4
1.0 BACKGROUND AND RATIONALE 9
1.1 Glioblastoma Multiforme 9
1.2 MRI-guided Laser Ablation (MLA) 9
1.3 Immune Dysregulation 11
1.4 MK-3475 11
1.5 Study Rationale 13
1.6 Correlative Studies Background 14
1.7 Rationale for Amendment 12 14
2.0 OBJECTIVES 15
2.1 Primary Objectives 15
2.2 Secondary Objectives 15
3.0 PATIENT SELECTION 15
3.1 Inclusion Criteria 15
3.2 Exclusion Criteria 17
3.3 Inclusion of Women and Minorities 19
4.0 REGISTRATION PROCEDURES 19
4.1 Confirmation of Patient Eligibility 20
4.2 Patient Registration in the Siteman Cancer Center OnCore Database 20
4.3 Assignment of UPN 20
4.4 Phase II Patients 21
5.0 TREATMENT PLAN 21
5.1 Premedication Administration 22
5.2 Agent Administration 22
5.3 MRI-Guided Laser Ablation 22
5.4 Phase I Dose Escalation Schema 23
5.5 Definition of MTD, DLT, and Dose Escalation Criteria 23
5.6 Toxicity, Response, and DLT Evaluations 24
5.7 General Concomitant Medication and Supportive Care Guidelines 25
5.8 Women of Childbearing Potential 26
5.9 Duration of Therapy 28
5.10 Second Course Phase 29
5.11 Duration of Follow-up 30
6.0 DOSE DELAYS/DOSE MODIFICATIONS 30
7.0 REGULATORY AND REPORTING REQUIREMENTS 34
7.1 Sponsor-Investigator Reporting Requirements 34
7.2 Reporting Requirements for Secondary Sites 38
7.3 Exceptions to Expedited Reporting 39
8.0 PHARMACEUTICAL INFORMATION 39
8.1 MK-3475 39
9.0 CORRELATIVE STUDIES 40
9.1 Blood Specimens 40
9.2 Cervical Lymph Node Samples - OPTIONAL 41
9.3 Tumor Tissue Specimens 42
10.0 STUDY CALENDAR 43
10.1 Phase I Study Calendar 43

10.2 Phase II Study Calendars.. 45
10.3 Second Course Phase Calendar 47
11.0 DATA SUBMISSION SCHEDULE.. 48
11.1 Adverse Event Collection in the Case Report Forms.. 48
12.0 MEASUREMENT OF EFFECT 49
12.1 Antitumor Effect – Solid Tumors... 49
12.2 Disease Parameters.. 51
12.3 Methods for Evaluation of Measurable Disease 51
13.0 DATA AND SAFETY MONITORING 54
14.0 AUDITING 56
15.0 STATISTICAL CONSIDERATIONS.. 57
15.1 Power Analysis.. 57
15.2 Analysis Plan.. 58
15.3 Phase II Stopping Rules... 58
15.4 Interim Analysis.. 58
15.5 Subgroup analysis 59
16.0 MULTICENTER REGULATORY REQUIREMENTS... 59
17.0 REFERENCES... 61
APPENDIX A: Karnofsky Performance Scale.. 64
APPENDIX B: Definitions for Adverse Event Reporting.. 65
APPENDIX C: Reporting Timelines 67
APPENDIX D: Washington University Serious Adverse Event Reporting Cover Sheet 70

1.0 BACKGROUND AND RATIONALE

1.1 Glioblastoma Multiforme

Glioblastoma multiforme (GBM) is the most common and lethal malignant brain tumor[1]. Despite advanced chemoradiotherapy, median survival is < 15 months, and < 5% of patients survive > 5 years[1-3]. Several targeted agents including monoclonal antibodies have been tested in GBM patients with minimal success[4], although these agents have shown anti-growth activity *in vitro*. This high failure rate is likely due to the redundancy in key growth pathways and the molecular and cellular heterogeneity within each tumor. Another explanation is the poor CNS penetration of these drugs due to the blood brain barrier (BBB). As a result, high drug doses were used to achieve therapeutic drug concentrations in the CNS, resulting in significant toxicities[4]. Thus an outstanding challenge in neuro-oncology has been to generate drugs that have excellent CNS penetration or methods that can compromise the BBB to enhance drug delivery.

1.2 MRI-guided Laser Ablation (MLA)

MRI-guided laser ablation (MLA) therapy for GBM is a novel minimally invasive surgery approved for cytoreductive treatment of brain tumors[5]. Dr. Eric Leuthardt and Dr. Albert Kim performed one of the first MLA procedures in the country and are experts in this technique. Currently Washington University has one of the largest clinical experiences using this new technology (Monteris, Winnipeg, CA), which has received FDA clearance for "use to ablate, necrotize, or coagulate soft tissue through interstitial irradiation or thermal therapy (LITT) in medicine and surgery in the discipline of neurosurgery with 1064 nm lasers," and is marketed under the brand name of NeuroBlate. MLA employs a small incision in the scalp and skull, through which a thin laser probe is inserted and guided by MR imaging to the core of a tumor mass where it delivers hyperthermic ablation from the core to the rim. The temperature at the core can reach 70°C, resulting in coagulative necrosis (Fig. 1). The

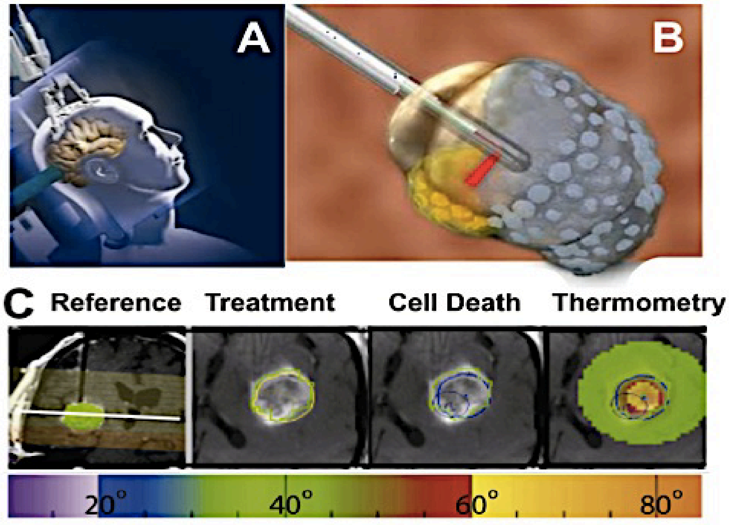

Figure 1: MRI-guided Heat Ablation Therapy (Monteris AutoLITT™). (A) Tripod base affixed to the skull with stereotactic wand visible. (B) Rendering of laser heating tumor from inside out. (C) Targeting strategy, treatment and cell death areas, and thermometry measurements. The target region was defines preoperatively based on MRI images (green sphere and green lines). Treatment areas were defines intraoperatively for each trajectory (yellow lines showing treatment area for trajectory #2). Cell death margins was determined as defined by trajectory and thermometry: thin and thick blue lines indicate cell death margins for trajectories #1 and #2, respectively. Thermometry measurements in degrees Celsius for the total treatment are plotted relative to the MRI.

temperature decrease in the peritumoral region but remains high enough (> 40°C) to induce disruption of the BBB as evidenced by new contrast enhancement in this region, while the original tumor enhancement is lost due to the heat ablation (Figs. 1 and 2).

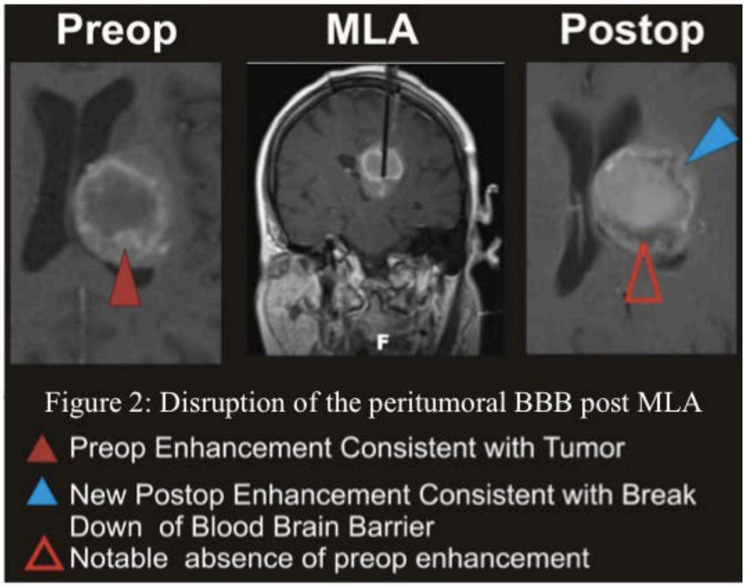

Figure 2: Disruption of the peritumoral BBB post MLA

General characteristics that make the lesion(s) favorable to treatment include the following: (1) the lesion(s) is (are) supratentorial and accessible from a cephalad approach (i.e., top one third of the head), (2) the lesion(s) is (are) unilateral, (3) the lesion(s) is (are) relatively well circumscribed, (4) the volume of lesion(s) can be encompassed by two 3-cm cylinders (i.e., 2 treatment trajectories), (5) a safe trajectory can be established relative to functional structures (i.e., eloquent cortex and corticospinal tract), and (6) the patient's body habitus can fit into the bore of the MRI. Trajectories are chosen to maximize lesion ablation and minimize the number of passes. MLA will be performed per FDA clearance for the NeuroBlate device using 1064nm lasers (for a detailed description of the procedure, please refer to these references[5-8]). Live intra-procedural treatment monitoring is critical for stereotactic LITT in order to assess for real-time tissue temperature and avoid treatment beyond the target zone. Fortunately, recent technological advances in intraoperative MR imaging when coupled with the NeuroBlate apparatus now enable accurate intraoperative thermal monitoring that is necessary for successful and safe ablation of intracranial lesions. Intra-procedural repetitive measurements of a T1-weighted 2-dimensional-FLASH sequence, or similar methods, provide temporally-sensitive thermometry measurements necessary to create controlled and conformal lesions (Fig. 1C).

The disrupted BBB may also allow release of tumor antigens into the circulation and influx of immune cells into the peritumoral region. In a series of patients treated with MLA at our institution, peritumor enhancement modestly increased 3 days after MLA while FLAIR signal changed only minimally, indicating that the post-MLA condition created peritumoral BBB disruption but early non-specific inflammatory edema was not a major feature. Interestingly, both peritumoral enhancement and FLAIR signal significantly increased 2 weeks after MLA and then persisted for several weeks. These delayed and persistent changes suggest that another effect of the BBB disruption after MLA could be the activation of an immune reaction leading to infiltration of tumor-specific lymphocytes and the resultant delayed inflammatory reaction.

1.2.1 Prior Clinical Experience with MLA

We have had one of the largest clinical experiences using MLA in the country. To date we have done more than 80 cases, of which approximately two-thirds are high-grade glial neoplasms. In regards to adverse events, patients have experienced transient aphasia, transient hemiparesis, transient hyponatremia, and a documented case of lower extremity deep venous thrombosis. Patients with aphasia or hemiparesis improved over time with steroid therapy. Hyponatremia resolved spontaneously. One patient experienced fatal meningitis. Subsequent in-depth analysis revealed that this patient's meningitis was due to an operating room infrastructure-related contamination and not due to equipment contamination related to performance of the MLA procedure.

1.3 Immune Dysregulation

Recent work has collectively demonstrated striking immune dysregulation in patients with GBM, including T cell lymphopenia and anergy, cytokine dysregulation, increased regulatory T cell (Treg) populations, and NK cell dysfunction, among others, which reflect immunologic compromise and functional impairment[9-12]. However, a growing list of potential tumor antigens has been identified, suggesting that tumor cell-specific recognition by immune cells may be biologically relevant and therapeutically exploitable[11,13]. Therefore, given the immune dysfunction characterized in GBM patients, approaches that potentiate the anti-glioma immune response are particularly exciting. A priori, because the immune system has evolved to recognize a tremendous diversity of antigenic epitopes in vertebrates, immune-potentiating efforts may be especially effective at targeting the heterogeneity that defines GBM. Recently, the success of antibody-based therapies targeting immune checkpoints[14,15] has generated field-wide enthusiasm that biological therapies may be broadly effect across many cancer types, including GBM. Of these agents, those targeting the negative regulator proteins CTLA-4 and PD-1/PD-L1 have shown clinical efficacy but have not been studied in GBM patients.

1.4 MK-3475

The importance of intact immune surveillance in controlling outgrowth of neoplastic transformation has been known for decades[16]. Accumulating evidence shows a correlation between tumor-infiltrating lymphocytes (TILs) in cancer tissue and favorable prognosis in various malignancies[17-21] . In particular, the presence of CD8+ T-cells and the ratio of CD8+ effector T-cells / FoxP3+ regulatory T-cells seems to correlate with improved prognosis and long-term survival in many solid tumors.

The PD-1 receptor-ligand interaction is a major pathway hijacked by tumors to suppress immune control. The normal function of PD-1, expressed on the cell surface of activated T-cells under healthy conditions, is to down-modulate unwanted or excessive immune responses, including autoimmune reactions. PD-1 (encoded by the gene Pdcd1) is an Ig superfamily member related to CD28 and CTLA-4 which has been shown to negatively regulate antigen receptor signaling upon engagement of its ligands (PD-L1 and/or

PD-L2)[22,23]. The structure of murine PD-1 has been resolved[24]. PD-1 and family members are type I transmembrane glycoproteins containing an Ig Variable-type (V-type) domain responsible for ligand binding and a cytoplasmic tail which is responsible for the binding of signaling molecules. The cytoplasmic tail of PD-1 contains 2 tyrosine-based signaling motifs, an immunoreceptor tyrosine-based inhibition motif (ITIM) and an immunoreceptor tyrosine-based switch motif (ITSM). Following T-cell stimulation, PD-1 recruits the tyrosine phosphatases SHP-1 and SHP-2 to the ITSM motif within its cytoplasmic tail, leading to the dephosphorylation of effector molecules such as CD3ζ, PKCθ and ZAP70 which are involved in the CD3 T-cell signaling cascade [22,25-27]. The mechanism by which PD-1 down modulates T-cell responses is similar to, but distinct from that of CTLA-4 as both molecules regulate an overlapping set of signaling proteins[28,29]. PD-1 was shown to be expressed on activated lymphocytes including peripheral CD4+ and CD8+ T-cells, B-cells, T regs and Natural Killer cells[30,31] . Expression has also been shown during thymic development on CD4-CD8- (double negative) T-cells as well as subsets of macrophages and dendritic cells [32] . The ligands for PD-1 (PD-L1 and PD-L2) are constitutively expressed or can be induced in a variety of cell types, including non-hematopoietic tissues as well as in various tumors [28,33-35] . Both ligands are type I transmembrane receptors containing both IgV- and IgC-like domains in the extracellular region and contain short cytoplasmic regions with no known signaling motifs. Binding of either PD-1 ligand to PD-1 inhibits T-cell activation triggered through the T-cell receptor. PD-L1 is expressed at low levels on various non-hematopoietic tissues, most notably on vascular endothelium, whereas PD-L2 protein is only detectably expressed on antigen-presenting cells found in lymphoid tissue or chronic inflammatory environments. PD-L2 is thought to control immune T-cell activation in lymphoid organs, whereas PD-L1 serves to dampen unwarranted T-cell function in peripheral tissues [28]. Although healthy organs express little (if any) PD-L1, a variety of cancers were demonstrated to express abundant levels of this T-cell inhibitor. PD-1 has been suggested to regulate tumor-specific T-cell expansion in subjects with melanoma (MEL) [36]. This suggests that the PD-1/PD-L1 pathway plays a critical role in tumor immune evasion and should be considered as an attractive target for therapeutic intervention.

Pembrolizumab is a potent and highly selective humanized monoclonal antibody (mAb) of the IgG4/kappa isotype designed to directly block the interaction between PD-1 and its ligands, PD-L1 and PD-L2. Keytruda™ (pembrolizumab) has recently been approved in the United Stated for the treatment of patients with unresectable or metastatic melanoma and disease progression following ipilimumab and, if BRAF V600 mutation positive, a BRAF inhibitor.

Please refer to the IB for preclinical and clinical data.

1.4.1 Rationale for Dosing

An open-label Phase I trial (Protocol 001) is being conducted to evaluate the safety and clinical activity of single agent MK-3475. The dose escalation portion of this trial evaluated three dose levels, 1 mg/kg, 3 mg/kg, and 10 mg/kg, administered every 2 weeks (Q2W) in subjects with advanced solid tumors. All three dose levels

were well tolerated and no dose-limiting toxicities were observed. This first in human study of MK-3475 showed evidence of target engagement and objective evidence of tumor size reduction at all dose levels (1 mg/kg, 3 mg/kg and 10 mg/kg Q2W). No MTD has been identified to date. 10.0 mg/kg Q2W, the highest dose tested in PN001, will be the dose and schedule utilized in Cohorts A, B, C and D of this protocol to test for initial tumor activity. Recent data from other clinical studies within the MK-3475 program has shown that a lower dose of MK-3475 and a less frequent schedule may be sufficient for target engagement and clinical activity.

PK data analysis of MK-3475 administered Q2W and Q3W showed slow systemic clearance, limited volume of distribution, and a long half-life (refer to IB). Pharmacodynamic data (IL-2 release assay) suggested that peripheral target engagement is durable (>21 days). This early PK and pharmacodynamic data provides scientific rationale for testing a Q2W and Q3W dosing schedule.

A population pharmacokinetic analysis has been performed using serum concentration time data from 476 patients. Within the resulting population PK model, clearance and volume parameters of MK-3475 were found to be dependent on body weight. The relationship between clearance and body weight, with an allometric exponent of 0.59, is within the range observed for other antibodies and would support both body weight normalized dosing or a fixed dose across all body weights. MK-3475 has been found to have a wide therapeutic range based on the melanoma indication. The differences in exposure for a 200 mg fixed dose regimen relative to a 2 mg/kg Q3W body weight-based regimen are anticipated to remain well within the established exposure margins of 0.5 – 5.0 for MK-3475 in the melanoma indication. The exposure margins are based on the notion of similar efficacy and safety in melanoma at 10 mg/kg Q3W vs. the proposed dose regimen of 2 mg/kg Q3W (i.e. 5-fold higher dose and exposure). The population PK evaluation revealed that there was no significant impact of tumor burden on exposure. In addition, exposure was similar between the NSCLC and melanoma indications. Therefore, there are no anticipated changes in exposure between different indication settings.

One of the potential immune-related adverse events of a PD-1 inhibitor is increased intracranial pressure due to edema resulting from drug-induced tumor-specific immune activation. In addition, mild edema can occasionally occur after MLA. Therefore, we have built in a dose escalation plan including a low dose of MK-3475 of 100 mg Q3W, an intermediate dose of 150 mg Q3W, and a high dose of 200 mg Q3W in order to determine the safety of this combination.

1.5 Study Rationale

The BBB is a major obstacle to drug delivery in the treatment of malignant brain tumors including GBM. Recent work also detailed immune dysregulation in patients with GBM, which is due in part to the BBB limiting tumor antigen presentation and access of immune

cells to the tumor. MLA has been noted to disrupt peritumoral BBB, which could then lead to increased access of new tumor antigens to the lymphovascular system and vice versa of immune effector cells to the tumor for effective activation of the immune system. Therefore, the combination of MK-3475 and MLA as proposed in this protocol is hypothesized to create a therapeutic synergy in which MLA increases material access to promote immune activation and then MK-3475 maximizes these tumor-specific immune reactions to impart effective tumor control.

1.6 Correlative Studies Background

In both arms of phase II, intratumoral PD-L1 expression will be determined by qRT-PCR and/or IHC. TIL profile will be assessed by flow and mass spectrometry or IHC to compare to the profile at initial surgery prior to recurrence (when available). Glioma-specific immune response will be assessed in cultured TIL, peripheral blood, and sampled cLN (where available). Pre- and post-surgery peripheral blood will be assessed by flow cytometry through the Center for Human Immunology and Immunotherapy immune monitoring core (for Washington University samples) and the Tran lab or the University of Florida Brain Tumor Immunotherapy Program (for the UF samples) for markers of T cell activation and anergy as well as cytokine profiles. In addition, PD-1-dependent signature in glioma cell-specific T cells from the peripheral blood and sampled cLN where samples and RNAseq are available can be generated by novel RNAseq methodology developed at Washington University and University of Florida in order to characterize the PD-1-dependent transcriptional signatures and develop a PD-1 dependent biomarker program that will be easily de-convoluted to provide data on serial changes in (a) cell type composition and (b) cell type activation programs. Furthermore, if necessary, serum or plasma will be assessed for the presence and level of soluble immune checkpoint molecules, cytokines, and chemokines using commercially available multiplex immunoassays.

1.7 Rationale for Amendment 12

Based on recent data[44-46], anti-PD1 antibody monotherapy given without or after surgical resection showed limited activity and failed to improve PFS or OS in recurrent GBM. Although this data was based on early staged small clinical trials, it is emerging knowledge in the field that due to the immunosuppressive nature and neoantigen access limitation of malignant gliomas that immune checkpoint inhibitors are likely inadequate when used alone. As determined during the May 2019 DSMC meeting, this has had an impact on the feasibility of completing enrollments in the MK-3475 monotherapy arm. Therefore, in the spirit of minimizing harm, potential or perceived, to subjects and to complete the study in a timely manner, patients will no longer be randomized in the phase II portion of this trial. After approval of Amendment 12, an additional 20 evaluable patients will be enrolled to participate in a single treatment arm with both MLA and MK-3475. Survival endpoints will be compared to historical data based on the above existing data.

2.0 OBJECTIVES

2.1 Primary Objectives

Phase I: To determine the maximal tolerated dose (MTD) of MK-3475 when combined with MLA for the treatment of recurrent malignant gliomas.

Phase II: To determine the progression-free survival (PFS) of patients with recurrent GBM, WHO grade IV being treated with MK-3475 plus MLA.

2.2 Secondary Objectives

1. Phase I: To determine the toxicity profile of MK-3475 in combination with MLA for the treatment of recurrent malignant gliomas.

2. Phase II: To determine the overall survival (OS) of patients with recurrent GBM, WHO grade IV being treated with MK-3475 plus MLA as compared to historical controls treated with MK-3475 alone.

3. Phase II: To assess the anti-glioma immune response before and after MK-3475 with MLA.

4. Phase II: To correlate intratumoral expression of PD-L1 and the frequency of glioma cell-specific cytotoxic T cells with PFS and OS.

5. Phase II: To identify PD-1-dependent biomarkers in glioma cell-specific T cells that negatively correlate with the frequency of glioma cell-specific cytotoxic T cells and PFS and OS.

3.0 PATIENT SELECTION

3.1 Inclusion Criteria

1. Phase I: Histologically confirmed grade III or IV malignant glioma.
   Phase II: Histologically confirmed grade IV malignant glioma (GBM).

   Note: GBM variants and secondary GBM, and suspected secondary GBM are allowed for both phase I and phase II.

2. Unequivocal evidence of tumor progression as documented by biopsy or brain MRI scan per RANO criteria (see Section 12).

3. There must be an interval of at least 12 weeks from the completion of standard frontline therapy to study registration unless there is unequivocal evidence for tumor recurrence per RANO criteria (see Section 12). When the interval is less than 12 weeks, the use

of perfusion imaging and/or PET scan is allowed to differentiate between unequivocal evidence of tumor recurrence and pseudoprogression. Standard frontline therapy is as described below:

a. For grade IV malignant gliomas (GBM): Standard frontline therapy for newly diagnosed GBM must include maximal feasible surgical resection (biopsy alone allowed), radiotherapy, and temozolomide chemotherapy. If the tumor was initially diagnosed as either a grade II or III tumor and now has recurred or progressed as a grade IV GBM, it will be considered a secondary recurrent grade IV GBM and will be eligible for this study as long as prior treatment included maximal feasible surgical resection (biopsy alone allowed), radiotherapy, and temozolomide chemotherapy.

b. For grade III malignant gliomas with 1p 19q codeletions: Standard frontline therapy for newly diagnosed grade III malignant gliomas must include maximal feasible surgical resection (biopsy alone allowed), radiotherapy, and chemotherapy (PCV or temozolomide). If the patient did not receive any or all components of the standard front line therapy as detailed above for newly diagnosed grade III gliomas with 1p 19q codeletions and the tumor then recurred or progressed, s/he must first receive at least one prior standard therapy or any appropriate combination of the components of standard therapy as detailed above and must experience further recurrence or progression before s/he is deemed eligible for this study. If the tumor was initially diagnosed as a grade II glioma with 1p 19q codeletions and now has recurred or progressed as a grade III tumor, it will be considered a secondary recurrent grade III glioma with 1p 19q codeletions and will be eligible for this study as long as prior treatment included maximal feasible surgical resection (biopsy alone allowed), radiotherapy, and chemotherapy (PCV or temozolomide).

c. For grade III malignant glioma without 1p 19q codeletions: Standard frontline therapy for newly diagnosed grade III malignant gliomas must include maximal feasible surgical resection (biopsy alone allowed), radiotherapy, and temozolomide chemotherapy. If the tumor was initially diagnosed as a grade II glioma without 1p 19q codeletions and now has recurred or progressed as a grade III tumor, it will be considered a secondary recurrent grade III glioma without 1p 19q codeletions and will be eligible for this study as long as prior treatment included maximal feasible surgical resection (biopsy alone allowed), radiotherapy, and temozolomide chemotherapy.

4. Candidate for MLA based on the size, location, and shape of the recurrent tumor as determined by the performing neurosurgeon. Surgical resection/debulking prior to MLA is allowed per standard of care but is not required; if the patient undergoes resection or debulking, it must have occurred at least 3 weeks prior to the first dose of MK-3475.

   For Phase II: if surgical resection/debulking prior to MLA is not indicated, a biopsy of the tumor will be done at the same time of MLA to obtain tumor tissue for both

diagnostic purposes and immune monitoring.

5. Patients who have undergone a resection for recurrence will be eligible. In those who have undergone a gross total resection, the MLA will be directed at treating a peritumoral margin of 0.5-1cm surrounding the resection cavity to disrupt the BBB and potentially increase access of MK-3475 to the peritumoral infiltrating glioma cells.

6. At least 18 years of age.

7. Karnofsky ≥ 60% (see Appendix A)

8. Normal bone marrow and organ function as defined below:
   a. ANC ≥ 1,500/mcL
   b. Platelets ≥ 100,000/mcL
   c. Hemoglobin ≥ 9 g/dL or ≥ 5.6 mmol/L
   d. Serum creatinine ≤ 1.5 x IULN OR creatinine clearance by Cockcroft-Gault ≥ 60 mL/min for patients with serum creatinine > 1.5 x IULN
   e. Serum total bilirubin ≤ 1.5 x IULN OR direct bilirubin ≤ IULN for patients with total bilirubin > 1.5 x IULN
   f. AST (SGOT) and ALT (SGPT) ≤ 2.5 x IULN
   g. INR or PT ≤ 1.5 x IULN unless patient is receiving anticoagulant therapy as long as PT or PTT is within therapeutic range of intended use of anticoagulants
   h. aPTT ≤ 1.5 x IULN unless patient is receiving anticoagulant therapy as long as PT or PTT is within therapeutic range of intended use of anticoagulants

9. Sexually active women of childbearing potential and men must agree to use contraceptive methods as described in Section 5.8 prior to study entry, for the duration of study participation, and for 120 days after last dose of MK-3475. Should a woman become pregnant or suspect she is pregnant while participating in this study, she must inform her treating physician immediately.

10. Patients with the ability to understand and willingness to sign an IRB approved written informed consent document will be enrolled into the trial. However, should a patient lose their ability to consent while participating in this study and s/he is still receiving clinical benefit from participation, s/he may continue on study with the consent of a Legally Authorized Representative.

3.2 Exclusion Criteria

1. Prior treatment with any anti-angiogenic agent (including bevacizumab).

2. Prior treatment with an anti-PD-1, anti-PD-L1, anti-PD-L2, **or** anti-CD137, or anti-cytotoxic T-lymphocyte-associated antigen-4 (CTLA-4) antibody (including ipilimumab or any other antibody or drug specifically targeting T-cell co-stimulation or checkpoint pathways).

3. Prior treatment with a monoclonal antibody within 4 weeks prior to the first dose of MK-3475 or has not recovered (i.e. ≤ grade 1 or at baseline) from adverse events due to agents administered more than 4 weeks earlier.

4. Prior chemotherapy, targeted small molecule therapy, or radiation therapy within 2 weeks prior to the first dose of MK-3475 or has not recovered (i.e. ≤ grade 1 or at baseline) from adverse events due to a previously administered agent.

   Note: patients with ≤ grade 2 neuropathy are an exception to this criterion and may qualify for the study.

   Note: if a patient underwent major surgery, s/he must have recovered adequately from the toxicity and/or complications from the intervention prior to starting therapy.

5. Candidate for curative resection or urgent surgical procedure(s) needed.

6. Presence of brainstem lesions or lesions that are less than 5 mm from the hypophysis or cranial nerves.

7. Multifocal gliomas that are bilateral. Patients with unilateral multifocal gliomas may be eligible if their multifocal disease can be treated effectively and safely in a single MLA procedure. Also note that corpus callosal tumors are eligible even if they are bilateral as long as they satisfy the size and shape limits of MLA as determined by the performing neurosurgeon.

8. Presence of leptomeningeal metastases.

9. Recent (within 8 weeks) history of CNS hemorrhage unless the hemorrhage is located within the tumor that will be removed en total during surgical debulking or ablated during MLA.

10. Requires therapeutic doses of anticoagulants unless anticoagulation can be safely discontinued before surgery per standard practice (e.g. first DVT for which anticoagulation has been at least 3 months and repeat imaging demonstrates resolution of DVT) or an IVC filter can be used in place of anticoagulation. Subjects are permitted to resume anticoagulation following surgery per discretion of treating physician and/or site SOPs.

11. Received prior local therapy (stereotactic radiosurgery, brachytherapy, or carmustine wafers) to the proposed area of MLA treatment.

12. Received a live vaccine or live-attenuated vaccine within 30 days prior to the first dose of MK-3475. Administration of killed vaccines is allowed.

13. Currently receiving any other investigational agents or has participated in a study of an investigational agent or using an investigational device within 3 weeks of the first dose

of MK-3475.

14. A history of allergic reactions attributed to compounds of similar chemical or biologic composition to MK-3475 or other agents used in the study.

15. Dexamethasone > 4 mg at the time of registration.

16. Has a diagnosis of immunodeficiency or is receiving chronic systemic steroid therapy (in dosing exceeding 10 mg daily of prednisone equivalent) or any other form of immunosuppressive therapy within 7 days prior to the first dose of trial treatment (with the exception of daily dexamethasone ≤ 4 mg).

17. Uncontrolled intercurrent illness including, but not limited to, ongoing or active infection, symptomatic congestive heart failure, unstable angina pectoris, cardiac arrhythmia, uncontrolled hypertension, or psychiatric illness/social situations that would limit compliance with study requirements.

18. Has an active autoimmune disease requiring systemic treatment within the past 2 years (i.e. with use of disease modifying agents, corticosteroids, or immunosuppressive drugs). Replacement therapy (e.g., thyroxine, insulin, or physiologic corticosteroid replacement therapy for adrenal or pituitary insufficiency, etc.) is not considered a form of systemic treatment.

19. Has a history of (non-infectious) pneumonitis/interstitial lung disease that required steroids or current pneumonitis/interstitial lung disease.

20. Pregnant and/or breastfeeding. Patient must have a negative serum or urine pregnancy test within 2 weeks of study entry.

21. Known history of hepatitis B (defined as hepatitis B surface antigen [HBsAg] reactive) or known active hepatitis C virus (defined as HCV RNA [qualitative] is detected) infection.

22. Known history of active TB (bacillus tuberculosis).

23. Known history of HIV (HIV 1/2 antibodies).

3.3 Inclusion of Women and Minorities

Both men and women and members of all races and ethnic groups are eligible for this trial.

4.0 REGISTRATION PROCEDURES

Patients must not start any protocol intervention prior to registration through the Siteman Cancer Center.

The following steps must be taken before registering patients to this study:

1. Confirmation of patient eligibility by Washington University
2. Registration of patient in the Siteman Cancer Center OnCore database
3. Assignment of unique patient number (UPN)

Once the patient has been entered in the Siteman Cancer Center OnCore database, the WUSM coordinator will forward verification of enrollment and the UPN via email.

4.1 Confirmation of Patient Eligibility

Confirm patient eligibility by collecting the information listed below and scanning and emailing it to the research coordinator listed in the *Siteman Cancer Center Clinical Trials Core Protocol Procedures for Secondary Sites* packet at least one business day prior to registering patient:

1. Your name and contact information (telephone number, fax number, and email address)
2. Your site PI's name, the registering MD's name, and your institution name
3. Patient's race, sex, and DOB
4. Three letters (or two letters and a dash) for the patient's initials
5. Currently approved protocol version date
6. Copy of signed consent form (patient name may be blacked out)
7. Planned date of enrollment
8. Completed eligibility checklist, signed and dated by a member of the study team
9. Copy of appropriate source documentation confirming patient eligibility

4.2 Patient Registration in the Siteman Cancer Center OnCore Database

Registrations may be submitted Monday through Friday between 8am and 5pm CT. Urgent late afternoon or early morning enrollments should be planned in advance and coordinated with the Washington University research coordinator. Registration will be confirmed by the research coordinator or his/her delegate by email within one business day. Verification of eligibility and registration should be kept in the patient chart.

All patients at all sites must be registered through the Siteman Cancer Center OnCore database at Washington University.

4.3 Assignment of UPN

Each patient will be identified with a unique patient number (UPN) for this study. All data will be recorded with this identification number on the appropriate CRFs.

4.4 Phase II Patients

As of Amendment 12, patients enrolled in the phase II portion of this trial will no longer be randomized. After registration and surgery (if applicable), these patients will undergo MLA followed by MK-3475 treatment.

5.0 TREATMENT PLAN

Patients enrolling in phase I will receive the first dose of MK-3475 will be given not more than 1 week after MLA, and it will be administered every 3 weeks +/- 4 days thereafter until progression or unacceptable toxicity.

The phase II dose will be determined during the phase I portion of this study. Patients enrolling in phase II will need to have tissue available for diagnostic purpose and for immunological monitoring. Surgical resection/debulking is per standard of care and optional for the purpose of this study and the performing neurosurgeon will determine whether each patient will undergo surgery.

All phase II patients will undergo MLA followed by MK-3475. Patients who are planning to have surgical resection/debulking will have MLA performed at least 3 weeks but not more than 6 weeks after surgical resection/debulking.

Patients who are not planning to have surgical resection/debulking will have a biopsy for tissue diagnosis and immune monitoring during MLA. If there is a patient with suspected GBM and the pathology results are not available by the time the patient is scheduled to start treatment with MK-3475, s/he will not be treated and will be removed from study. “Determined to be ineligible” will be listed as the “Reason Off Therapy” in the OnCore database. The date pathology results were reviewed and/or the date the final determination about eligibility was made will be used as the “Date Off Therapy.” Patients removed from study due to suspected GBM not being confirmed through pathology will not be included in the patient total enrolled and will be replaced.

All patients will receive their first dose of MK-3475 not more than 1 week after MLA (if applicable) or biopsy, and then every 3 weeks +/- 4 days thereafter. For patients who had surgical debulking but did not undergo MLA, MK-3475 will be started at least 3 weeks but not more than 6 weeks after surgical debulking, and then every 3 weeks +/- 4 days thereafter. Dosing of MK-3475 will continue at the dose determined by phase I until progression or unacceptable toxicity.

For all phase II patients, research blood will be drawn prior to MLA and immediately prior to the first dose of MK-3475. Subsequently, research blood will be drawn at doses 2, 4, 6, 9, 18, 36, and at the end of treatment. In addition, an optional ipsilateral cervical lymph node may be sampled by FNA prior to or at the time of surgical procedure (including surgical debulking, MLA, or biopsy) and again 6 weeks after surgery. Those patients who, in the event of surgical intervention for clinical reasons of a radiographically progressing tumor, undergo a subsequent surgical procedure (whether biopsy or resection) will have tissue collected for correlative analysis after histopathologic diagnosis of recurrent GBM.

Additionally, for patients enrolled to phase II who have surgical intervention for a radiographically progressing tumor but subsequent surgical pathology demonstrating a predominant immune-mediated reaction **with no obvious progression of tumor**, such subjects will be deemed not to have progressed and be allowed to resume assigned study treatment at a time deemed safe by the treating physician, but no later than 8 weeks from the surgery.

5.1 Premedication Administration

No premedications are required, but antiemetics may be given as per institutional practice if needed.

5.2 Agent Administration

MK-3475 will be given intravenously over the course of 30 minutes (-5 min/+10 min) on an outpatient basis. The starting dose for the phase I portion of the study is 100 mg every 3 weeks; please refer to the dose escalation schema in Section 5.4 for dosing during phase I. As determined during the phase I portion of the study, the phase II dose of MK-3475 for all patients is 200 mg. In the phase I portion of this study, MK-3475 will be given every 3 weeks starting no more than 1 week after MLA. In the phase II portion of this study, MK-3475 will be given every 3 weeks beginning no more than 1 week (+/- 3 days) after MLA.

5.3 MRI-Guided Laser Ablation

MLA is a minimally invasive laser surgery, which employs a small incision in the scalp and skull, through which a thin laser probe is inserted and guided by MR imaging to the core of the tumor mass where it delivers hyperthermic ablation from the core to the rim. The maximal temperature in the core can reach 70°C, resulting in coagulative necrosis. MLA will be performed as per standard of care. In those who have undergone a gross total resection, the MLA will be directed at treating a peritumoral margin of 0.5-1cm surrounding the resection cavity to disrupt the BBB and potentially increase access of MK-3475 to the peritumoral infiltrating glioma cells.

After initiation of anesthesia and placement of an MRI-compatible head holder, a MRI-guided navigation system is registered using preoperative MRI to define the centroid of the target lesion. After inserting the probe in the lesion and positioning the patient in the MRI scanner, the laser (1064 nm wavelength, 10-14 Watts power, pulse duration 2.2 seconds on, 1.7 seconds off) is activated and real-time thermal isodose MRI is monitored to achieve sufficient treatment (no limit to exposure duration). The NeuroBlate software provides predictive thermal dosage lines. Yellow lines for 43 °C are set for 2 min and blue lines for 43°C for 10 min to estimate ablation volume. These thermal dosage lines will be used to establish treatment duration, movement of the probe, and additional trajectories depending on the location, shape and size of the target lesion. For some patients, additional trajectories are chosen preoperatively; for other patients, additional trajectories are chosen based on thermal dosage lines of the initial trajectory. After successful ablation, the probe is removed and the wound is closed in routine fashion. Patients are generally hospitalized for one or

two days after the procedure, and monitoring will take place as described below.

Perioperative care will be consistent with current standard care for patients undergoing craniotomy or biopsy of a brain tumor. This includes either ICU or step-down unit post-operative stay. Typically, these patients are placed on perioperative steroids to reduce the risk of post-treatment edema. Also, the patients are placed on prophylactic antiepileptics (i.e. Keppra) to mitigate perioperative and post-treatment seizures. During post-operative recovery, patients have routine neurological exams per the care team. In the ICU this is on an hourly basis; in the stepdown unit this is every two hours. Additionally, post-operative labs are obtained immediately after surgery and on the following day to ensure there are no metabolic abnormalities. They are also seen in the morning and in the late afternoon by the neurosurgical team.

5.4 Phase I Dose Escalation Schema

**Dose Escalation Schedule**	
**Dose Level**	**MK-3475 Dose**
Level 1 (Starting Dose)	100 mg IV q3wks
Level 2	150 mg IV q3wks
Level 3	200 mg IV q3wks

A decision regarding dose escalation will not take place until all patients in the Level 1 cohort have received the post-MLA dose of MK-3475 and the Principal Investigator has been able to review all toxicities for 3 weeks after that dose.

NOTE: No later than two weeks following the completion of the first two months of treatment for the first 3 patients enrolled in this trial, interim safety data must be provided to the FDA as a formal IND submission. This submission should include a listing of all AEs of grade 2 or greater severity observed. For all AEs of grade 3 or greater severity, and for all SAEs, a narrative description of the events should be included, along with the timing of the event in relationship to study treatment, interventions, duration, and outcome.

5.5 Definition of MTD, DLT, and Dose Escalation Criteria

5.5.1 Definition of Maximum Tolerated Dose (MTD)

The maximum tolerated dose (MTD) is defined as the dose level immediately below the dose level at which 2 patients of a cohort (of 2 to 6 patients) experience dose-limiting toxicity during the first cycle. Dose escalations will proceed until the MTD has been reached or, failing that, 200 mg q3wks will be considered the MTD.

5.5.2 Dose Limiting Toxicities (DLTs)

DLT is defined as any of the following that occur during the time frame between the first dose of MK-3475 and 3 weeks after the second dose of MK-3475 that are attributed as possibly, probably, or definitely related to the study treatment:

- Grade 2 or greater diarrhea
- Autoimmune hypophysitis
- Grade 3 or greater hepatitis
- Grade 2 or greater pneumonitis
- Significant intracranial edema requiring high-dose steroid (defined as > 16 mg/day and/or inability to taper steroids to ≤ 8 mg/day within 4 weeks due to recurrent symptoms attributable to excessive cranial edema)
- Grade 3 or greater non-hematologic toxicity
- Grade 3 or greater hematologic toxicity

5.5.3 Dose Escalation Criteria

Dose escalations will proceed as follows after the occurrence of DLT:

Number of Patients with DLT at a Given Dose Level	Escalation Decision Rule
0 out of 3	Enter 3 patients at the next dose level
2 or 3 out of 3	Dose escalation will be stopped and 3 patients will be entered at the next lowest dose level if only 3 patients were treated previously at that dose.
1 out of 3	• Enter at least 3 more patients at this dose level.<br>• If 0 of these 3 patients experience DLT, proceed to the next dose level.<br>• If 1 or more of this group suffer DLT, then dose escalation is stopped and 3 additional patients will be entered at the next lowest dose level if only 3 patients were treated previously at that dose.
≤ 1 out of 6 at highest dose level below the maximally administered dose	This is generally the recommended phase II dose. At least 6 patients must be entered at the recommended phase II dose.

5.6 Toxicity, Response, and DLT Evaluations

All patients who receive any treatment with MK-3475 are evaluable for toxicity. Patients are evaluated from first receiving study treatment until a 30-day follow-up after the conclusion of treatment or death.

All phase II patients are evaluable for disease response unless they come off study before any disease assessment.

A phase I patient is evaluable for DLT assessment from the time of the first dose of MK-3475 through 3 weeks following the second dose of MK-3475. Once the patient has received his/her third dose of MK-3475, s/he will no longer be evaluated for DLTs. Patients who do not incur a DLT and who receive only a single dose of MK-3475 or who do not undergo MLA should be considered not evaluable for DLT and should be replaced.

5.7 General Concomitant Medication and Supportive Care Guidelines

All treatments that the investigator considers necessary for a patient's welfare may be administered at the discretion of the investigator in keeping with the community standards of medical care.

Patients are prohibited from receiving the following therapies while in screening for and enrolled in this trial:

- Anti-cancer systemic chemotherapy or biological therapy. Treatment with bevacizumab as a steroid-sparing agent is allowed on trial.
- Immunotherapy not specified in this protocol
- Chemotherapy not specified in this protocol
- Investigational agents other than MK-3475
- Radiation therapy
- Live vaccines within 30 days prior to the first dose of treatment and while participating in the trial. Examples of live vaccines include, but are not limited to, the following: measles, mumps, rubella, varicella/zoster, yellow fever, rabies, BCG, and typhoid vaccine.

5.7.1 Supportive Care Guidelines for Infusion Reactions

Pembrolizumab may cause severe or life-threatening reactions including severe hypersensitivity or anaphylaxis. Signs and symptoms usually develop during or shortly after drug infusion and generally resolve completely within 24 hours of completion of infusion.

The table below shows treatment guidelines for patients who experience an infusion reaction associated with administration of MK-3475.

NCI CTCAE Grade	Treatment	Premedication at subsequent dosing
<u>Grade 1</u><br>Mild reaction; infusion interruption not indicated; intervention not indicated	Increase monitoring of vital signs as medically indicated until the subject is deemed medically stable in the opinion of the investigator.	None
<u>Grade 2</u><br>Requires infusion interruption but responds promptly to symptomatic treatment (e.g., antihistamines, NSAIDS, narcotics, IV fluids); prophylactic medications indicated for < =24 hrs	**Stop Infusion and monitor symptoms.**<br>Additional appropriate medical therapy may include but is not limited to: IV fluids, antihistamines, NSAIDS, acetaminophen, Narcotics<br><br>Increase monitoring of vital signs as medically indicated until the subject is deemed medically stable in the opinion of the investigator.<br>If symptoms resolve within one hour of stopping drug infusion, the infusion may be restarted at 50% of the original infusion rate (e.g. from 100 mL/hr to 50 mL/hr). Otherwise dosing will be held until symptoms resolve and the subject should be premedicated for the next scheduled dose.<br><br>**Subjects who develop Grade 2 toxicity despite adequate premedication should be permanently discontinued from further trial treatment administration.**	Subject may be premedicated 1.5h (± 30 minutes) prior to infusion of MK-3475 with:<br><br>Diphenhydramine 50 mg po (or equivalent dose of antihistamine).<br><br>Acetaminophen 500-1000 mg po (or equivalent dose of antipyretic).

NCI CTCAE Grade	Treatment	Premedication at subsequent dosing
<u>Grades 3 or 4</u><br>Grade 3:<br>Prolonged (i.e., not rapidly responsive to symptomatic medication and/or brief interruption of infusion); recurrence of symptoms following initial improvement; hospitalization indicated for other clinical sequelae (e.g., renal impairment, pulmonary infiltrates)<br><br>Grade 4:<br>Life-threatening; pressor or ventilatory support indicated	**Stop Infusion.**<br>Additional appropriate medical therapy may include but is not limited to: IV fluids, antihistamines, NSAIDS, acetaminophen, narcotics, oxygen, pressors, corticosteroids, epinephrine<br><br>Increase monitoring of vital signs as medically indicated until the subject is deemed medically stable in the opinion of the investigator.<br>Hospitalization may be indicated.<br><br>**Subject is permanently discontinued from further trial treatment administration.**	No subsequent dosing
Appropriate resuscitation equipment should be available in the room and a physician readily available during the period of drug administration.		

5.8 Women of Childbearing Potential

Women of childbearing potential (defined as women with regular menses, women with amenorrhea, women with irregular cycles, women using a contraceptive method that precludes withdrawal bleeding, and women who have had a tubal ligation) are required to have a negative serum or urine pregnancy test within 72 hours prior to the first dose of MK-3475.

Pembrolizumab may have adverse effects on a fetus in utero.

For this trial, male subjects will be considered to be of non-reproductive potential if they have azoospermia (whether due to having had a vasectomy or due to an underlying medical condition).

A woman is considered fertile following menarche and until becoming post-menopausal unless permanently sterile (see below). Women in the following categories are not considered of childbearing potential:

- Premenarchal
- Premenopausal female with 1 of the following:
  - Documented hysterectomy
  - Documented bilateral salpingectomy
  - Documented bilateral oophorectomy
    Note: Documentation can come from the site personnel's review of the participant's medical records, medical examination, or medical history interview.

- Postmenopausal female
  - A postmenopausal state is defined as no menses for 12 months without an alternative medical cause.
    - A high follicle stimulating hormone (FSH) level in the postmenopausal range may be used to confirm a postmenopausal state in women not using hormonal contraception or hormonal replacement therapy (HRT). However,

in the absence of 12 months of amenorrhea, confirmation with two FSH measurements in the postmenopausal range is required.

- Females on HRT and whose menopausal status is in doubt will be required to use one of the non-hormonal highly effective contraception methods if they wish to continue their HRT during the study. Otherwise, they must discontinue HRT to allow confirmation of postmenopausal status before study enrollment.

Contraception Requirements

Subjects should be informed that taking the study medication may involve unknown risks to the fetus (unborn baby) if pregnancy were to occur during the study. In order to participate in the study subjects of childbearing potential must adhere to the contraception requirement (described below) from the day of study medication initiation (or 14 days prior to the initiation of study medication for oral contraception) throughout the study period up to 120 days after the last dose of trial therapy. If there is any question that a subject of childbearing potential will not reliably comply with the requirements for contraception, that subject should not be entered into the study.

Male Participants:

Male participants with female partners of childbearing potential are eligible to participate if they agree to one of the following during the protocol defined timeframe:

- Be abstinent from penile-vaginal intercourse as their usual and preferred lifestyle (abstinent on a long term and persistent basis) and agree to remain abstinent
- Use a male condom plus partner use of a contraceptive method with a failure rate of <1% per year as described in the table below when having penile-vaginal intercourse with a woman of childbearing potential who is not currently pregnant.
    - Note: Men with a pregnant or breastfeeding partner must agree to remain abstinent from penile-vaginal intercourse or use a male condom during each episode of penile penetration.

Female Participants

Female participants of childbearing potential are eligible to participate if they agree to use a highly effective method of contraception consistently and correctly as described in the table below during the protocol-defined timeframe.

**Highly Effective Contraceptive Methods That Are User Dependent [a]**<br>*Failure rate of < 1% per year when used consistently and correctly.*
● Combined (estrogen- and progestogen-containing) hormonal contraception [b, c]<br>○ Oral<br>○ Intravaginal<br>○ Transdermal<br>○ Injectable
● Progestogen-only hormonal contraception [b, c]<br>○ Oral<br>○ Injectable
**Highly Effective Methods That Have Low User Dependency**<br>*Failure rate of <1% per year when used consistently and correctly.*

• Progestogen- only contraceptive implant [b, c]<br>• Intrauterine hormone-releasing system (IUS) [b]<br>• Intrauterine device (IUD)<br>• Bilateral tubal occlusion
• **Vasectomized partner**<br>A vasectomized partner is a highly effective contraception method provided that the partner is the sole male sexual partner of the WOCBP and the absence of sperm has been confirmed. If not, an additional highly effective method of contraception should be used.
• **Sexual abstinence**<br>Sexual abstinence is considered a highly effective method only if defined as refraining from heterosexual intercourse during the entire period of risk associated with the study treatment. The reliability of sexual abstinence needs to be evaluated in relation to the duration of the study and the preferred and usual lifestyle of the participant.)
Notes:<br>Use should be consistent with local regulations regarding the use of contraceptive methods for participants of clinical studies.<br><br>a) Typical use failure rates are lower than perfect-use failure rates (i.e. when used consistently and correctly).<br>b) If hormonal contraception efficacy is potentially decreased due to interaction with study treatment, condoms must be used in addition to the hormonal contraception during the treatment period and for at least [X days, corresponding to time needed to eliminate study treatment plus 30 days for study treatments with genotoxic potential] after the last dose of study treatment.<br>c) If locally required, in accordance with Clinical Trial Facilitation Group (CTFG) guidelines, acceptable hormonal contraceptives are limited to those which inhibit ovulation.

Pregnancy Testing

WOCBP should only be included after a negative highly sensitive urine or serum pregnancy test.

Following initiation of treatment, pregnancy testing will be performed whenever an expected menstrual cycle is missed or when pregnancy is otherwise suspected, after the last dose of study treatment, and as required locally.

Pregnancy testing will be performed whenever an expected menstrual cycle is missed or when pregnancy is otherwise suspected.

5.9 Duration of Therapy

If at any time the constraints of this protocol are considered to be detrimental to the patient’s health and/or the patient no longer wishes to continue protocol therapy, the protocol therapy should be discontinued and the reason(s) for discontinuation documented in the case report forms.

In the absence of treatment delays due to adverse events, treatment may continue for up to 24 months (35 cycles; the number of treatments is calculated starting with the first dose) or until one of the following criteria applies:

- Documented and confirmed disease progression
- Death
- Adverse event(s) that, in the judgment of the investigator, may cause severe or permanent harm or which rule out continuation of study drug
- General or specific changes in the patient's condition render the patient unacceptable for further treatment in the judgment of the investigator
- Suspected pregnancy
- Serious non-compliance with the study protocol
- Lost to follow-up
- Patient withdraws consent
- Investigator removes the patient from study
- The Siteman Cancer Center decides to close the study

Patients who prematurely discontinue treatment for any reason will be followed as indicated in the study calendar.

Patients currently on pembrolizumab beyond the 2 year/35 cycle limit at the time of the approval of Amendment 13 may continue to receive pembrolizumab unless there is progression, toxicity or agreement by the patient and PI to come off therapy.

Participants who discontinue pembrolizumab after receiving 35 doses are eligible for retreatment with pembrolizumab if they progress during follow-up provided they meet the requirements detailed in Section 5.10. Participants may receive an additional 17 cycles (12 months) of pembrolizumab during the Second Course Phase (Retreatment).

5.10 Second Course Phase

All participants who discontinue treatment with pembrolizumab with at least stable disease are eligible for 17 further cycles (approximately 1 year) of pembrolizumab if they progress during follow-up. This retreatment is termed the Second Course Phase of this study and is only available if all of the following conditions are satisfied:

1. The study is open at the time the patient qualifies for retreatment.
2. The patient experienced investigator-determined radiographic disease progression by RANO criteria (see Section 12) during the follow-up period after stopping initial treatment with pembrolizumab and:
    a. No new anticancer treatment was administered after the last dose of pembrolizumab and
    b. The participant meets all of the inclusion criteria for lab parameters and performance status
3. The patient EITHER:
    a. Had stable disease, partial response, or complete response based on RANO criteria and discontinued pembrolizumab after completion of 35 doses (approximately 2 years) for reasons other than disease progression or intolerability or
    b. Discontinued pembrolizumab after attaining an investigator-determined

confirmed complete response by RANO criteria, received at least 8 cycles of pembrolizumab prior to discontinuation, and received at least 2 treatments with pembrolizumab beyond the date when initial CR was declared

An objective response or disease progression that occurs during the Second Course Phase for a participant will not be counted as an event for the primary analysis of either endpoint in this study.

5.11 Duration of Follow-up

Patients will have one follow-up visit 4 weeks (+/- 1 week) after stopping treatment. They will then be followed for up to 2 years for survival. Routine follow-up will be conducted as per standard of care but is not mandated by this protocol. Patients removed from study for unacceptable adverse events will be followed until resolution or stabilization of the adverse event.

6.0 DOSE DELAYS/DOSE MODIFICATIONS

AEs associated with pembrolizumab exposure may represent an immunologic etiology. These immune-related AEs (irAEs) may occur shortly after the first dose or several months after the last dose of pembrolizumab treatment and may affect more than on body system simultaneously. Therefore, early recognition and initiation of treatment is critical to reduce complications.

Based on existing clinical study data, most irAEs were reversible and could be managed with interruptions of pembrolizumab, administration of corticosteroids and/or other supportive care. For suspected irAEs, ensure adequate evaluation to confirm etiology or exclude other causes. Additional procedures or tests such as bronchoscopy, endoscopy, skin biopsy may be included as part of the evaluation. Based on the severity of irAEs, withhold or permanently discontinue pembrolizumab and administer corticosteroids. Dose modification and toxicity management guidelines for irAEs associated with pembrolizumab are provided in the table below.

General instructions:

1. Corticosteroid taper should be initiated upon AE improving to Grade 1 or less and continue to taper over at least 4 weeks.
2. For situations where pembrolizumab has been withheld, pembrolizumab can be resumed after AE has been reduced to Grade 1 or 0 and corticosteroid has been tapered. Pembrolizumab should be permanently discontinued if AE does not resolve within 12 weeks of last dose or corticosteroids cannot be reduced to ≤ 10 mg prednisone or equivalent per day within 12 weeks.
3. For severe and life-threatening irAEs, IV corticosteroid should be initiated first followed by oral steroid. Other immunosuppressive treatment should be initiated if irAEs cannot be controlled by corticosteroids.

**Immune-related AEs**	**Toxicity grade or conditions (CTCAEv4.0)**	**Action taken to pembrolizumab**	**irAE management with corticosteroid and/or other therapies**	**Monitor and follow-up**
Pneumonitis	Grade 2	Withhold	• Administer corticosteroids (initial dose of 1 to2mg/kg prednisone or equivalent) followed by taper. Add prophylactic antibiotics for opportunistic infections	• Monitor participants for signs and symptoms of pneumonitis<br>• Evaluate participants with suspected pneumonitis with radiographic imaging and initiate corticosteroid treatment<br>•
	Grade 3 or 4, or recurrent grade 2	Permanently discontinue		
Diarrhea / colitis	Grade 2 or 3	Withhold	• Administer corticosteroids (initial dose of 1 to2mg/kg prednisone or equivalent) followed by taper	• Monitor participants for signs and symptoms of enterocolitis (i.e. diarrhea, abdominal pain, blood or mucus in stool with or without fever) and of bowel perforation (i.e. peritoneal signs and ileus).<br>• Participants with ≥ Grade 2 diarrhea suspecting colitis should consider GI consultation and performing endoscopy to rule out colitis.<br>• Participants with diarrhea/colitis should be advised to drink liberal quantities of clear fluids. If sufficient oral fluid intake is not feasible, fluid and electrolytes should be substituted via IV infusion.
	Grade 4 or recurrent Grade 3	Permanently discontinue		
AST / ALT elevation or Increased Bilirubin	Grade 2[a]	Withhold	• Administer corticosteroids (initial dose of 0.5 to1mg/kg prednisone or equivalent) followed by taper	• Monitor with liver function tests (consider weekly or more frequently until liver enzyme value returned to baseline or is stable)
	Grade 3[b] or 4[c]	Permanently discontinue	• Administer corticosteroids (initial dose of 1to 2mg/kg prednisone or equivalent) followed by taper	

Type 1 diabetes mellitus (T1DM) or Hyperglycemia	Newly onset T1DM or Grade 3 or 4 hyperglycemia associated with evidence of β-cell failure	Withhold[d]	• Initiate insulin replacement therapy for participants with T1DM<br>• Administer anti-hyperglycemic in participants with hyperglycemia	• Monitor participants for hyperglycemia or other signs and symptoms of diabetes.
Hypophysitis	Grade 2	Withhold	• Administer corticosteroids and initiate hormonal replacements as clinically indicated.	• Monitor for signs and symptoms of hypophysitis (including hypopituitarism and adrenal insufficiency)
	Grade 3 or 4	Withhold or permanently discontinue[d]		
Hyperthyroidism	Grade 2	Continue	• Treat with nonselective beta-blockers (e.g. propranolol) or thionamides as appropriate	• Monitor for signs and symptoms of thyroid disorders.
	Grade 3 or 4	Withhold or Permanently discontinue[d]		
Hypothyroidism	Grade 2, 3, or 4	Continue	• Initiate thyroid replacement hormones (e.g. levothyroxine or liothyroinine) per standard of care	• Monitor for signs and symptoms of thyroid disorders.
Nephritis : grading according to increased creatinine or acute kidney injury	Grade 2	Withhold	• Administer corticosteroids (prednisone 1 to2mg/kg or equivalent) followed by taper.	• Monitor changes of renal function
	Grade 3 or 4	Permanently discontinue		
Neurological Toxicities	Grade 2	Withhold	• Based on severity of AE administer corticosteroids	• Ensure adequate evaluation to confirm etiology and/or exclude other causes
	Grade 3 or 4	Permanently discontinue		
Myocarditis	Grade 1	Withhold	• Based on severity of AE administer corticosteroids	• Ensure adequate evaluation to confirm etiology and/or exclude other causes
	Grade 2, 3 or 4	Permanently discontinue		
Exfoliative Dermatologic Conditions	Suspected SJS, TEN, or DRESS	Withhold	• Based on severity of AE administer corticosteroids	• Ensure adequate evaluation to confirm etiology or exclude other causes
	Confirmed SJS, TEN, or DRESS	Permanently discontinue		
All Other irAEs	Persistent Grade 2	Withhold	• Based on severity of AE administer corticosteroids	• Ensure adequate evaluation to confirm etiology or exclude other causes
	Grade 3	Withhold or discontinue based on the type of event[e]		
	Grade 4 or recurrent Grade 3	Permanently discontinue		

[a]AE(s)=adverse event(s); ALT= alanine aminotransferase; AST=aspartate aminotransferase; CTCAE=Common Terminology Criteria for Adverse Events; DRESS=Drug Rash with Eosinophilia and Systemic Symptom; GI=gastrointestinal; IO=immuno-oncology; ir=immune related; IV=intravenous; SJS=Stevens-Johnson Syndrome; T1DM=type 1 diabetes mellitus; TEN=Toxic Epidermal Necrolysis; ULN=upper limit of normal.

Note: Non-irAE will be managed as appropriate, following clinical practice recommendations.

[a] AST/ALT: >3.0 to 5.0 x ULN if baseline normal; >3.0 to 5.0 x baseline, if baseline abnormal;
bilirubin:>1.5 to 3.0 x ULN if baseline normal; >1.5 to 3.0 x baseline if baseline abnormal

[b] AST/ALT: >5.0 to 20.0 x ULN, if baseline normal; >5.0 to 20.0 x baseline, if baseline abnormal; bilirubin:>3.0 to 10.0 x ULN if baseline normal; >3.0 to 10.0 x baseline if baseline abnormal

[c] AST/ALT: >20.0 x ULN, if baseline normal; >20.0 x baseline, if baseline abnormal;
bilirubin: >10.0 x ULN if baseline normal; >10.0 x baseline if baseline abnormal

[d] The decision to withhold or permanently discontinue pembrolizumab is at the discretion of the investigator or treating physician. If control achieved or ≤ Grade 2, pembrolizumab may be resumed.

[e] Events that require discontinuation include, but are not limited to: encephalitis and other clinically important irAEs (eg, vasculitis and sclerosing cholangitis).

For information on the management of adverse events, see Section 5.7.

7.0 REGULATORY AND REPORTING REQUIREMENTS

The entities providing oversight of safety and compliance with the protocol require reporting as outlined below. Please refer to Appendix B for definitions and Appendix C for a grid of reporting timelines.

Adverse events will be tracked from start of treatment through 30 days following the last day of study treatment. SAEs will be tracked for 90 days following the last dose of study treatment. All adverse events must be recorded on the toxicity tracking case report form. This includes all adverse events:

- belonging to the category "Nervous System Disorders" in the CTCAE version 4.03, regardless of attribution
- experienced by the patient during the administration of MK-3475, regardless of attribution
- that are CTCAE version 4.03 gradable laboratory values, regardless of attribution

The only adverse events that will not be recorded are adverse events that are clearly related to MLA and/or surgery AND ALSO not related to MK-3475.

Refer to the data submission schedule in Section 11 for instructions on the collection of AEs in the EDC.

Reporting requirements for Washington University study team may be found in Section 7.1. Reporting requirements for secondary site study teams participating in Washington University-coordinated research may be found in Section 7.3.

7.1 Sponsor-Investigator Reporting Requirements

7.1.1 Reporting to the Human Research Protection Office (HRPO) at Washington University

Reporting will be conducted in accordance with Washington University IRB Policies.

Pre-approval of all protocol exceptions must be obtained prior to implementing the change.

7.1.2 Reporting to the Quality Assurance and Safety Monitoring Committee (QASMC) at Washington University

The Washington University Sponsor-Investigator (or designee) is required to notify the QASMC of any unanticipated problem involving risks to participants or others occurring at WU or any BJH or SLCH institution that has been reported to and acknowledged by HRPO. (Unanticipated problems reported to HRPO and withdrawn during the review process need not be reported to QASMC.)

QASMC must be notified within **10 days** of receipt of IRB acknowledgment via

email to qasmc@wustl.edu. Submission to QASMC must include the myIRB form and any supporting documentation sent with the form.

For events that occur at secondary sites, the Washington University Sponsor Investigator (or designee) is required to notify the QASMC within 10 days of Washington University notification via email to qasmc@wustl.edu. Submission to QASMC must include either the myIRB form and supporting documentation or (if not submitted to myIRB) the date of occurrence, description of the event, whether the event is described in the currently IRB approved materials, the event outcome, determination of relatedness, whether currently enrolled participants will be notified, and whether the informed consent document and/or any study procedures will be modified as a result of this event.

7.1.3 Reporting to Secondary Sites

The Washington University Sponsor-Investigator (or designee) will notify the research team at each secondary site of all unanticipated problems involving risks to participants or others that have occurred at other sites within **10 working days** of the occurrence of the event or notification of the Sponsor-Investigator (or designee) of the event. This includes events that take place both at Washington University and at other secondary sites, if applicable.

7.1.4 Reporting to the FDA

The conduct of the study will comply with all FDA safety reporting requirements. **PLEASE NOTE THAT REPORTING REQUIREMENTS FOR THE FDA DIFFER FROM REPORTING REQUIREMENTS FOR HRPO/QASMC.** It is the responsibility of the Washington University principal investigator to report any unanticipated problem to the FDA as follows:

- Report any unexpected fatal or life-threatening suspected adverse reaction (refer to Appendix B for definitions) no later than 7 calendar days after initial receipt of the information.
- Report a suspected adverse reaction that is both serious and unexpected (SUSAR, refer to Appendix B) no later than 15 calendar days after it is determined that the information qualifies for reporting. Report an adverse event (refer to Appendix B) as a suspected adverse reaction only if there is evidence to suggest a causal relationship between the drug and the adverse event, such as:
  - A single occurrence of an event that is uncommon and known to be strongly associated with drug exposure
  - One or more occurrences of an event that is not commonly associated with drug exposure but is otherwise uncommon in the population exposed to the drug
  - An aggregate analysis of specific events observed in a clinical trial that indicates those events occur more frequently in the drug treatment group

than in a concurrent or historical control group

- Report any findings from epidemiological studies, pooled analysis of multiple studies, or clinical studies that suggest a significant risk in humans exposed to the drug no later than 15 calendar days after it is determined that the information qualifies for reporting.
- Report any findings from animal or in vitro testing that suggest significant risk in humans exposed to the drug no later than 15 calendar days after it is determined that the information qualifies for reporting.
- Report any clinically important increase in the rate of a serious suspected adverse reaction of that listed in the protocol or IB within 15 calendar days after it is determined that the information qualifies for reporting.

Submit each report as an IND safety report in a narrative format or on FDA Form 3500A or in an electronic format that FDA can process, review, and archive. Study teams must notify the Siteman Cancer Center Protocol Development team of each potentially reportable event within 1 business day after initial receipt of the information, and must bring the signed 1571 and FDA Form 3500A to the Siteman Cancer Center Protocol Development team no later than 1 business day prior to the due date for reporting to the FDA.

Each notification to FDA must bear prominent identification of its contents ("IND Safety Report") and must be transmitted to the review division in the Center for Drug Evaluation and Research (CDER) or in the Center for Biologics Evaluation and Research (CBER) that has responsibility for review of the IND. Relevant follow-up information to an IND safety report must be submitted as soon as the information is available and must be identified as such ("Follow-up IND Safety Report").

7.1.5 Reporting to Merck & Co., Inc.

The WU PI shall forward to Merck's Global Safety group (FAX 215-661-6229) any SAE and SUSAR information, including, but not limited to, all initial and follow-up information involving any study subject in the study within 24 hours (whichever comes first) of learning of the SAE or SUSAR.

"Adverse Event" or "AE" shall mean any untoward medical occurrence in a Study subject who is administered the Study Drug regardless of whether or not a causal relationship with the Study Drug exists. By way of example and without limitation, an AE can be any unfavorable and unintended sign (for example, an abnormal laboratory finding), symptom, or disease temporally associated with the use of the Study Drug.

"Serious Adverse Event" or "SAE" shall mean any untoward medical occurrence in a Study subject who is administered the Study Drug that results in death, a life-threatening drug experience, requires inpatient hospitalization or prolongation of existing hospitalization, results in persistent or significant disability/incapacity, or

is a congenital anomaly/birth defect, cancer, or is a new cancer if the cancer is the condition of the study, or overdose. Other important medical events that may jeopardize the patient or may require intervention to prevent one of the outcomes listed previously should also be considered "serious."

"Suspected Unexpected Serious Adverse Reaction" or "SUSAR" shall mean any Serious Adverse Event, the nature, severity or frequency of which is not consistent with information in the most current investigator's brochure, or with respect to a marketed product the most current Summary of Product Characteristics (SPC) or Package Insert.

7.1.5.1 Reporting Overdose

For purposes of this trial, an overdose of pembrolizumab will be defined as any dose of 1,000 mg or greater (≥5 times the indicated dose). No specific information is available on the treatment of overdose of pembrolizumab. Appropriate supportive treatment should be provided if clinically indicated. In the event of overdose, the subject should be observed closely for signs of toxicity. Appropriate supportive treatment should be provided if clinically indicated.

If an adverse event(s) is associated with ("results from") the overdose of a Merck product, the adverse event(s) is reported as a serious adverse event, even if no other seriousness criteria are met.

If a dose of Merck's product meeting the protocol definition of overdose is taken without any associated clinical symptoms or abnormal laboratory results, the overdose is reported as a non-serious Event of Clinical Interest (ECI), using the terminology "accidental or intentional overdose without adverse effect."

All reports of overdose with and without an adverse event must be reported within 24 hours to the Sponsor and within 2 working days to Merck Global Safety. (Attn: Worldwide Product Safety; FAX 215 661-6229)

7.1.5.2 Reporting Events of Clinical Interest

Selected non-serious and serious adverse events are also known as Events of Clinical Interest (ECI) and must be reported within 2 working days to Merck Global Safety by the WU PI. (Attn: Worldwide Product Safety; FAX 215 661-6229)

Events of clinical interest are defined in Appendix B.

7.1.5.3 Reporting Pregnancy and Lactation

Although pregnancy and infant exposure during breastfeeding are not considered adverse events, it is the responsibility of investigators or their designees to report any pregnancy or lactation in a subject (spontaneously reported to them) that occurs during the trial.

Pregnancies and infant exposures during breastfeeding that occur after the consent form is signed but before registration must be reported by the investigator if they cause the subject to be excluded from the trial, or are the result of a protocol-specified intervention, including but not limited to washout or discontinuation of usual therapy, diet, placebo treatment or a procedure.

Pregnancies and infant exposures during breastfeeding that occur from the time of registration through 120 days following cessation of Sponsor's product, or 30 days following cessation of treatment if the subject initiates new anticancer therapy, whichever is earlier, must be reported by the investigator. All reported pregnancies must be followed to the completion/termination of the pregnancy. Pregnancy outcomes of spontaneous abortion, missed abortion, benign hydatidiform mole, blighted ovum, fetal death, intrauterine death, miscarriage and stillbirth must be reported as serious events (Important Medical Events). If the pregnancy continues to term, the outcome (health of infant) must also be reported.

Such events must be reported within 24 hours to the Sponsor and within 2 working days to Merck Global Safety. (Attn: Worldwide Product Safety; FAX 215 661-6229)

7.2 Reporting Requirements for Secondary Sites

The research team at each secondary site is required to promptly notify the Washington University Sponsor-Investigator and Designee of all serious adverse events (Refer to Appendix B) within **1 working day** of the occurrence of the event or notification of the secondary site's PI of the event. This notification may take place via email if there is not yet enough information for a formal written report using a FDA form 3500a (MedWatch form) and Washington University cover sheet (Appendix D). A formal written report must be sent to the Washington University Sponsor-Investigator within 4 **calendar days** (for fatal or life-threatening suspected adverse reactions**)** or 11 calendar days (for serious unexpected adverse reactions) of the occurrence of the event or notification of the secondary site's PI of the event.

The research team at a secondary site is responsible for following its site's guidelines for reporting applicable events to its site's IRB according to its own institutional guidelines. The research team at Washington University is responsible for reporting all applicable events to the FDA and Merck & Co. as needed.

Washington University pre-approval of all protocol exceptions must be obtained prior to implementing the change. Local IRB approval must be obtained as per local guidelines. Washington University IRB approval is not required for protocol exceptions occurring at secondary sites.

7.3 Exceptions to Expedited Reporting

Events that do not require expedited reporting as described in Section 7.1 include:

- Planned hospitalizations
- Hospitalizations < 24 hours
- Respite care
- Events related to disease progression

Events that do not require expedited reporting must still be captured in the EDC.

8.0 PHARMACEUTICAL INFORMATION

8.1 MK-3475

8.1.1 MK-3475 Description

MK-3475 is a potent humanized IgG4 mAb with high specificity of binding to the PD-1 receptor, thus inhibiting its interaction with PD-L1 and PD-L2. Based on preclinical in vitro data, MK-3475 has high affinity and potent receptor blocking activity for PD-1. MK-3475 has an acceptable preclinical safety profile and is being advanced for clinical development as an IV immunotherapy for advanced malignancies.

8.1.2 Clinical Pharmacology

Refer to Section 5.2 of the IB.

8.1.3 Pharmacokinetics and Drug Metabolism

Refer to Section 5.2 of the IB.

8.1.4 Supplier

MK-3475 will be provided free of charge by Merck & Co., Inc.

8.1.5 Dosage Form and Preparation

Merck will provide MK-3475 as a liquid drug product.

8.1.6 Storage and Stability

MK-3475 should be stored under refrigerated conditions (2°C - 8°C).

If not used immediately, vials and/or IV bags may be stored at 2-8 °C for up to a cumulative time of 20 hours. If refrigerated, the vials and/or IV bags should be allowed to equilibrate to room temperature prior to subsequent use. MK-3475 solutions may be stored at room temperature for a cumulative time of up to 4 hours.

8.1.7 Administration

MK-3475 will be given intravenously over the course of 30 minutes (-5 min/+10 min) on an outpatient basis.

8.1.8 Special Handling Instructions

No special handling instructions.

9.0 CORRELATIVE STUDIES

The collections for correlative studies are for patients enrolled in phase II only unless otherwise indicated.

9.1 Blood Specimens

9.1.1 Collection of Specimens

Forty mL of blood will be collected into 4 pink top tubes (with EDTA) for isolation of PBMCs at the following time points **for Phase II patients only**:

- No more than one week prior to surgical procedure (including resection/debulking, MLA or biopsy)
- No more than 3 days prior to the first dose of MK-3475
- No more than 7 days prior to the second dose of MK-3475 and at doses 4, 6, 9, 18, and 36
- At the end of treatment visit

9.1.2 Handling of Specimens

One 10 mL vial will be used for isolation of plasma via centrifugation. The remaining vials will be used for isolation of peripheral blood mononuclear cells (PBMCs) using Ficoll per standard protocol[37,38].

9.1.3 Shipping of Specimens

Blood samples collected at WUSM should be transported to and stored in Albert

Kim's lab (located at Couch Biomedical Research Building 4515 McKinley Ave, Room 6110) or the Siteman Cancer Center's Tissue Procurement Core (located on the 5th floor of the BJCIH) at ambient temperature immediately after collection and processed immediately upon arrival. If a delay in transport or processing of greater than 1 hour is anticipated, samples must be stored on ice. All attempts must be used to process samples within 4 hours of collection.

Blood samples collected at UF should be transported to Dr. Tran's lab to be processed within their immunophenotyping lab at ambient temperature immediately after collection and processed immediately upon arrival. If a delay in transport or processing of greater than 1 hour is anticipated, samples must be stored on ice. All attempts must be used to process samples within 4 hours of collection.

Preliminary processing of the scRNAseq samples will be completed by the MGI lab locally at WUSM. To ensure data analysis consistency, the resulting data will then be stripped of any identifying information and shared with UF, where it will be aggregated and analyzed as a cohort.

9.2 Cervical Lymph Node Samples - OPTIONAL

9.2.1 Collection of Specimens

Ipsilateral cervical lymph nodes of all phase II patients will be sampled by FNA (regardless of whether the node is pathologically enlarged) at the following time points:

- Time of surgical resection/debulking or biopsy or MLA/biopsy
- 6 weeks +/- 1 week after surgical resection/debulking or biopsy or MLA/biopsy (if tumor-specific precursor frequency in PBMC is low)

9.2.2 Handling of Specimens

Lymph nodes will be minced and cells isolated using a cell strainer per standard protocol (REF). Suspended lymph node lymphocytes will be Ficoll separated and the mononuclear cell population (PBMC) isolated per standard protocol[37,38]

9.2.3 Shipping of Specimens

Lymph node samples collected at WUSM should be placed in a buffered solution (e.g. PBS, a tissue culture media), transported to and stored in Albert Kim's lab (located at Couch Biomedical Research Building 4515 McKinley Ave, Room 6110) at ambient temperature, and processed immediately upon arrival. If a delay in transport or processing of greater than 1 hour is anticipated, samples must be placed on ice. All attempts must be used to process samples within 4 hours of collection.

Lymph node samples collected at UF should be placed a buffered solution (e.g. PBS, a tissue culture media), transported to Dr. Tran's lab in the

immunophenotyping lab at ambient temperature, and processed immediately upon arrival. If a delay in transport or processing of greater than 1 hour is anticipated, samples must be placed on ice. All attempts must be used to process samples within 4 hours of collection.

9.3 Tumor Tissue Specimens

9.3.1 Collection of Specimens

Any tissue (from either surgical debulking or biopsy or MLA/biopsy) available after histopathologic diagnosis of GBM will be collected for correlated studies as follows:

- Flash frozen for DNA and RNA isolation and for priming of DC
- Fixed and embedded in paraffin for IHC
- Acutely dissociated to generate patients' primary GBM cell lines and tumor infiltrating lymphocyte culture if adequate tissue is available.

Phase II patients will have tissue collected.

Those patients who, in the event of surgical intervention for clinical reasons of a radiographically progressing tumor, undergo a subsequent surgical procedure (whether biopsy or resection) will be collected after histopathologic diagnosis of progressive GBM.

9.3.2 Handling of Specimens

Tissue cryopreservation and paraffin embedding are per standard protocol. One half of the tissue will be equally divided into 2 parts: one part will be flash frozen and the other part will be fixed and embedded in paraffin (FFPE). FFPE samples are to be batch shipped to a MERCK-designated laboratory for central performance of PD-L1 IHC. Five (5) unstained slides may be submitted in lieu of the FFPE tissue block. The other half of the tissue will be divided into 2 parts: one part will be acutely dissociated by digestion with collagenase and hyaluronidase for patient-derived glioma cell line generation, and the other part will be minced into 1 mm chunks then plated for tumor-infiltrating lymphocyte cultures per standard protocol.

9.3.3 Shipping of Specimens

Brain tumor samples collected at WUSM should be placed in a buffered solution (e.g. PBS, a tissue culture media), transported to the laboratory of Albert Kim (3rd floor of McMillan building), or Siteman Cancer Center Tissue Procurement Core at ambient temperature and processed immediately upon arrival. From there the tissue will be divided. The half that will be used for the flash frozen stock and FFPE blocks will be transported and processed in the Siteman Cancer Center's Tissue Procurement Core. The other half that will be used to generate dissociated cells will be delivered immediately to Dr. Kim's lab (contact person: Tanner Johanns,

tannerjohanns@wustl.edu). If a delay in transport or processing of greater than 1 hour is anticipated, samples must be stored on ice. All attempts must be used to process samples within 4 hours of collection.

Brain tumor samples collected at UF should be placed in a buffered solution (e.g. PBS, a tissue culture media), transported to the Florida Center for Brain Tumor Research (FCBTR) tumor bank at UF at ambient temperature and processed immediately upon arrival. From there the tissue will be divided. The half that will be used for the flash frozen stock and FFPE blocks will be processed in the FCBTR. The other half that will be used to generate dissociated cells will be delivered to Dr. Tran's lab immediately (contact person: Alexandra Sherman, Alexandra.sherman@neurosurgery.ufl.edu. If a delay in transport or processing of greater than 1 hour is anticipated, samples must be stored on ice. All attempts must be used to process samples within 4 hours of collection.

10.0 STUDY CALENDAR

10.1 Phase I Study Calendar

	Screening	Day 1	Day 2 to Day 8	Q3W[9]	EOT	Follow-Up[2]
H&P, KPS	X		X	X	X	
Brain MRI	X			X[4]		
CBC	X		X[8]	X		
CMP[7]	X		X[8]	X		
INR, PT/PTT	X					
βhCG[6]	X		X[5]			
TSH	X			X[1]		
MK-3475			X	X[3]		
MLA		X[8]				
AE assessment	X -- X					

1. To be checked at every odd-numbered dose of MK-3475
2. There will be one follow-up visit 4 weeks after EOT, and then patients will be followed for up to 2 years for survival
3. Treatment can continue for a maximum of 2 years/35 cycles after MLA. Patients currently on MK-3475 beyond the 2 year/35 cycle limit at the time of Amendment 13 may remain on MK-3475 unless there is progression, toxicity, or agreement by the patient and PI to discontinue treatment. Refer to Section 5.10 for guidance on whether a patient qualifies for re-treatment in the Second Course Phase and refer to Section 10.3 for the Second Course Phase calendar.
4. Every 9 weeks +/- 1 week
5. No more than 72 hours prior to the first dose. If the screening pregnancy test is performed within that window, only one pregnancy test is required.
6. Women of childbearing potential only
7. CMP consists of albumin, alk phos, ALT, AST, BUN, Ca++, Cl-, CO2, creatinine, glucose, K+, Na+, total bili, total protein
8. Patients will receive peri- and post-operative care as per SOC. This typically includes treatment with steroids and anti-epileptics, repeated neurological exams (approx. hourly in the ICU and approx. every 2 hours in the step-down unit), and draws for CBC and CMP immediately post-MLA and again the following day.

9. +/- 4 days

10.2 Phase II Study Calendars

10.2.1 MK-3475 + MLA

	Screening	Surgical Resection/ Debulking (OPTIONAL)	Day 1 (MLA) (no surgical resection / debulking)[15]	Day 1 (MLA) (3 weeks post-surgical resection / debulking[16])	Day 2 to Day 9	Q3W[17]	EOT[3]
H&P, KPS	X				X[12]	X	X
Brain MRI	X				X[20]	X[9]	
CBC	X[18]				X	X	
CMP[13]	X[18]				X	X	
INR, PT/PTT	X[18]						
βhCG[11]	X[18]				X[10]		
TSH	X[18]				X[2]	X[2]	
MK-3475					X[1]	X[4]	
MLA			X[6, 15]	X[6, 16, 19]			
Surgical resection (optional)		X					
Biopsy			X[14]				
Research blood		X[5,7]	X[5, 7]		X[5]	X[5]	X[5]
Research lymph nodes		X[7]	X[7]			X[8]	
Research tissue		X[7]	X[7]				
AE assessment	X---X[21]						

1. To begin no more than 1 week (+/- 3 days) after MLA or 3 weeks after surgery.
2. To be checked at the first MK-3475 dose and then every other dose thereafter
3. There will be one follow-up visit 4 weeks after EOT, and then patients will be followed for up to 2 years for survival
4. Patients currently on MK-3475 beyond the 2 year/35 cycle limit at the time of this amendment may remain on MK-3475 unless there is progression, toxicity or agreement by the patient and PI to come off therapy. The number of treatments is calculated starting with the first dose. Participants who stop the combination or MK-3475 after receiving 35 doses may be eligible for retreatment if they progress after stopping study intervention provided they meet the requirements detailed in Section 5.10. Participants may be retreated in the Second Course Phase (Retreatment) for up to an additional 17 cycles (approximately 1 year).
5. Refer to Section 9.1
6. Patients will receive peri- and post-operative care as per SOC. This typically includes treatment with steroids and anti-epileptics, repeated neurological exams (approx. hourly in the ICU and approx. every 2 hours in the step-down unit), and draws for CBC and CMP immediately post-MLA and again the following day.
7. At time of surgery
8. 6 weeks post-surgery only (+/- 1 week)
9. Every 9 weeks (+/- 1 week)
10. No more than 72 hours prior to the first dose only. Additional pregnancy tests are not required.
11. Women of childbearing potential only
12. One week +/- 2 days following MLA or on the first dose of MK-3475 (whichever comes first)
13. CMP consists of albumin, alk phos, ALT, AST, BUN, Ca++, Cl-, CO2, creatinine, glucose, K+, Na+, total bili, total protein.
14. For tissue diagnosis and immune monitoring. Biopsy is only for patients who did not have surgical resection and will be performed during MLA.
15. Applies only to patients who did not have surgical resection.
16. Applies only to patients who had surgical resection.
17. +/- 4 days
18. Within 2 weeks prior to registration.
19. MLA will take place at least 3 weeks but not more than 6 weeks after surgical resection/debulking.
20. An MRI should be collected and evaluated after any surgical procedure (MLA, surgical resection/debulking, or biopsy).
21. Adverse events will be followed for 30 days following last treatment. SAEs will be tracked for 90 days following the last dose of study treatment.

10.2.2 MK-3475 – MLA

This arm is not available to newly enrolled patients as of Amendment 12.

	Screening	Surgical Resection/ Debulking (OPTIONAL)[14]	Biopsy Only[15]	Cycle 1 Day 1[11]	Q3W[16]	EOT[3]
H&P, KPS	X			X[11]	X	X
Brain MRI	X			X[18]	X[8]	
CBC	X[17]			X	X	
CMP[12]	X[17]			X	X	
INR, PT/PTT	X[17]					
βhCG[10]	X[17]			X[9]		
TSH	X[17]			X[2]	X[2]	
MK-3475				X[1]	X[4]	
Surgical resection (optional)		X				
Biopsy			X[13]			
Research blood		X[5,6]	X[5,6]	X[5]	X[5]	X[5]
Research lymph nodes		X[6]	X[6]		X[7]	
Research tissue		X[6]	X[6]			
AE assessment	X--X[19]					

1. To begin no more than 1 week (+/- 3 days) after biopsy (if applicable) or 3 weeks after surgery.
2. To be checked at the first MK-3475 dose and then every other dose thereafter
3. There will be one follow-up visit 4 weeks after EOT, and then patients will be followed for up to 2 years for survival
4. Treatment can continue for a maximum of 2 years/35 cycles after MLA. Patients currently on MK-3475 beyond the 2 year/35 cycle limit at the time of Amendment 13 may remain on MK-3475 unless there is progression, toxicity, or agreement by the patient and PI to discontinue treatment. Refer to Section 5.10 for guidance on whether a patient qualifies for re-treatment in the Second Course Phase and refer to Section 10.3 for the Second Course Phase calendar.
5. Refer to Section 9.1
6. At time of surgery
7. 6 weeks post-surgery only (+/- 1 week)
8. Every 9 weeks (+/- 1 week)
9. No more than 72 hours prior to the first dose only. Additional pregnancy tests are not required.
10. Women of childbearing potential only
11. One week +/- 2 days following biopsy or on the first dose of MK-3475 (whichever comes first). For patients who underwent surgical resection/debulking, MK-3475 will be started at least 3 weeks but not more than 6 weeks after surgery.
12. CMP consists of albumin, alk phos, ALT, AST, BUN, Ca++, Cl-, CO2, creatinine, glucose, K+, Na+, total bili, total protein.
13. For tissue diagnosis and immune monitoring. Biopsy is only for patients who did not have surgical resection and for patients in which the treating physician deems a biopsy clinically necessary.
14. Applies only to patients who had surgical resection.
15. Applies only to patients who did not have surgical resection.
16. +/- 4 days
17. Within 2 weeks prior to registration. As of amendment 12, patients will no longer be randomized in the Phase II portion of this trial. Phase II patients enrolled after the approval of amendment 12 will participate in a single treatment arm with MK-3475 and MLA and follow the schedule of events in section 10.2.1 of the protocol.
18. A standard of care MRI after the biopsy procedure is optional at the discretion of the treating neurosurgeon.
19. Adverse events will be followed for 30 days following last treatment. SAEs will be tracked for 90 days following the last dose of study treatment.

10.3 Second Course Phase Calendar

Day 1 of Second Course must start no more than 4 weeks after disease progression.

	**Screening**	**Day 1**	**Q3W[10]**	**EOT[8]**
H&P, KPS	X	X	X	X
Brain MRI	X		X[6]	
CBC	X[3]	X	X	
CMP[1]	X[3]	X	X	
INR, PT/PTT	X[3]			
βhCG[2]	X[3]	X[4]		
TSH	X[3]	X[5]	X[5]	
MK-3475		X	X[7]	
AE assessment	X--X[9]			

1. CMP consists of albumin, alk phos, ALT, AST, BUN, Ca++, Cl-, CO2, creatinine, glucose, K+, Na+, total bili, total protein.
2. Women of childbearing potential only
3. Within 4 weeks of progression.
4. No more than 72 hours prior to the first dose only. Additional pregnancy tests are not required.
5. To be checked at the first MK-3475 dose and then every other dose thereafter
6. Every 9 weeks (+/- 1 week)
7. Participants may be retreated in the Second Course Phase (Retreatment) for up to an additional 17 cycles (approximately 1 year).
8. There will be one follow-up visit 4 weeks after EOT, and then patients will be followed for up to 2 years for survival
9. Adverse events will be followed for 30 days following last treatment. SAEs will be tracked for 90 days following the last dose of study treatment.
10. +/- 4 days

11.0 DATA SUBMISSION SCHEDULE

Case report forms with appropriate source documentation will be completed according to the schedule listed in this section.

Case Report Form	Submission Schedule
Original Consent Form	Prior to registration
Registration Form<br>Eligibility Form<br>Demographics Form<br>Medical Record Number Form<br>Participant Address Form<br>On-Study Form<br>On-Study Treatment History Form<br>On-Study Surgical History Form	Prior to starting treatment
MK-3475 Dosing Form	With each dose of MK-3475 (during main study and second course as applicable)
MLA Form	Time of MLA
Surgery Form<br>Treatment Assignment Form	Time of surgery
Research Nodes and Tissue Form (Phase II only)	Time of surgical procedure<br>6 weeks post-surgical procedure
Research Blood Form (Phase II only)	Prior to starting treatment<br>Time of MLA<br>Time of surgical procedure<br>With each dose of MK-3475 (Per section 9.1)
Retreatment Form	Time of start of second course
Adverse Events Form	Continuous
Treatment Summary Form	Completion of treatment
Follow Up Form	Per protocol
Imaging Form	Baseline, every 9 +/-1 weeks, and end of treatment
MedWatch Form	See Section 7.0 for reporting requirements

Any queries generated by Washington University must be responded to within 28 days of receipt by the participating site. The Washington University research team will conduct a regular review of data status at all secondary sites, with appropriate corrective action to be requested as needed.

11.1 Adverse Event Collection in the Case Report Forms

All adverse events that occur beginning with start of treatment (minus exceptions defined in Section 1.0) must be captured in the Toxicity Form. Baseline AEs should be captured on the Medical History Form.

Participant death due to disease progression should be reported on the Toxicity Form as grade 5 disease progression. If death is due to an AE (e.g. cardiac disorders: cardiac arrest),

report as a grade 5 event under that AE. Participant death must also be recorded on the Death Form.

12.0 MEASUREMENT OF EFFECT

12.1 Antitumor Effect – Solid Tumors

For the purposes of this study, patients should be re-evaluated for response every 9 +/- 1 weeks. In addition to a baseline scan, confirmatory scans should also be obtained 9 weeks (not less than 4) weeks following initial documentation of objective response.

Response and progression will be evaluated in this study using the updated response assessment criteria for high-grade gliomas: Response Assessment in Neuro-Oncology (RANO) working group guideline [JCO 28(11): 1963-1972, 2010].

Criteria for Determining First Progression Depending on Time from Initial Chemoradiotherapy

First Progression	Definition
Progressive disease < 12 weeks after completion of chemoradiotherapy	Progression can only be defined using diagnostic imaging if there is new enhancement outside of the radiation field (beyond the high-dose region or 80% isodose line) or if there is unequivocal evidence of viable tumor on histopathologic sampling (eg, solid tumor areas [ie, > 70% tumor cell nuclei in areas], high or progressive increase in MIB-1 proliferation index compared with prior biopsy, or evidence for histologic progression or increased anaplasia in tumor). Note: Given the difficulty of differentiating true progression from pseudoprogression, clinical decline alone, in the absence of radiographic or histologic confirmation of progression, will not be sufficient for definition of progressive disease in the first 12 weeks after completion of concurrent chemoradiotherapy.
Progressive disease ≥ 12 weeks after chemoradiotherapy completion	1. New contrast-enhancing lesion outside of radiation field on decreasing, stable, or increasing doses of corticosteroids.<br>2. Increase by ≥ 25% in the sum of the products of perpendicular diameters between the first postradiotherapy scan, or a subsequent scan with smaller tumor size, and the scan at 12 weeks or later on stable or increasing doses of corticosteroids.<br>3. Clinical deterioration not attributable to concurrent medication or comorbid conditions is sufficient to declare progression on current treatment but not for entry onto a clinical trial for recurrence.<br>4. For patients receiving antiangiogenic therapy, significant increase in T2/FLAIR nonenhancing lesion may also be considered progressive disease. The increased T2/FLAIR must have occurred with the patient on stable or increasing doses of corticosteroids compared with baseline scan or best response after initiation of therapy and not be a result of comorbid events (eg, effects of radiation therapy, demyelination, ischemic injury, infection, seizures, postoperative changes, or other treatment effects).

Criteria for Response Assessment Incorporating MRI and Clinical Factors (Adapted from JCO 2010)

Response	Criteria
Complete response	• Requires all of the following: complete disappearance of all enhancing measurable and nonmeasurable disease sustained for at least 4 weeks.<br>• No new lesions; stable or improved nonenhancing (T2/FLAIR) lesions.<br>• Patients must be off corticosteroids (or on physiologic replacement doses only) and stable or improved clinically. Note: Patients with nonmeasurable disease only cannot have a complete response; the best response possible is stable disease.
Partial response	Requires all of the following:<br>• ≥ 50% decrease compared with baseline in the sum of products of perpendicular diameters of all measurable enhancing lesions sustained for at least 4 weeks.<br>• No progression of nonmeasurable disease.<br>• Stable or improved nonenhancing (T2/FLAIR) lesions on same or lower dose of corticosteroids compared with baseline scan; the corticosteroid dose at the time of the scan evaluation should be no greater than the dose at time of baseline scan.<br>• Stable or improved clinically. Note: Patients with nonmeasurable disease only cannot have a partial response; the best response possible is stable disease.
Stable disease	Requires all of the following:<br>• Does not qualify for complete response, partial response, or progression.<br>• Stable nonenhancing (T2/FLAIR) lesions on same or lower dose of corticosteroids compared with baseline scan. In the event that the corticosteroid dose was increased for new symptoms and signs without confirmation of disease progression on neuroimaging, and subsequent follow-up imaging shows that this increase in corticosteroids was required because of disease progression, the last scan considered to show stable disease will be the scan obtained when the corticosteroid dose was equivalent to the baseline dose.
Progression	Defined by any of the following:<br>• ≥ 25% increase in sum of the products of perpendicular diameters of enhancing lesions compared with the smallest tumor measurement obtained either at baseline (if no decrease) or best response, on stable or increasing doses of corticosteroids*. The absolute increase in any dimension must be at least 5mm when calculating the products.<br>• Significant increase in T2/FLAIR nonenhancing lesion on stable or increasing doses of corticosteroids compared with baseline scan or best response after initiation of therapy* not caused by comorbid events (e.g. radiation therapy, demyelination, ischemic injury, infection, seizures, postoperative changes, or other treatment effects).

Response	Criteria
	• Any new measureable lesion.<br>• Clear clinical deterioration not attributable to other causes apart from the tumor (e.g. seizures, medication adverse effects, complications of therapy, cerebrovascular events, infection, and so on) or changes in corticosteroid dose.<br>• Failure to return for evaluation as a result of death or deteriorating condition; or clear progression of nonmeasurable disease.

- NOTE. All measurable and nonmeasurable lesions must be assessed using the same techniques as at baseline.
- Abbreviations: MRI, magnetic resonance imaging; FLAIR, fluid-attenuated inversion recovery.
- Stable doses of corticosteroids include patients not on corticosteroids.

12.2 Disease Parameters

Measurable disease: Bi-dimensionally measurable lesions with clearly defined margins by MRI scan of ≥ 10 mm. All tumor measurements must be recorded in millimeters (or decimal fractions of centimeters).

Non-measurable or evaluable disease: Uni-dimensionally measurable lesions or lesions with margins not clearly defined such as areas of T2/FLAIR signal abnormality or poorly defined enhancing abnormality.

Note: For cystic lesions, the only measurable part is any enhancement area around the cyst that is clearly defined and bi-dimensionally measurable. The cyst itself should not be considered measurable or non-measureable disease.

Target lesions: All measurable lesions that are seen at baseline MRI. Target lesions should be selected on the basis of their size (lesions with the longest diameter), but in addition should be those that lend themselves to reproducible repeated measurements. It may be the case that, on occasion, the largest lesion does not lend itself to reproducible measurement in which circumstance the next largest lesion, which can be measured reproducibly should be selected. When there are too many measurable lesions, choose the largest 3 lesions as target lesions to follow. The other measurable lesions should be considered evaluable for the purpose of objective status determination.

Non-target lesions: All non-measurable lesions should be identified as non-target lesions and should also be recorded at baseline. Measurements of these lesions are not required, but the presence, absence, or in rare cases unequivocal progression of each should be noted throughout follow-up.

12.3 Methods for Evaluation of Measurable Disease

All measurements should be taken and recorded in metric notation using a ruler. All baseline MRI evaluations should be performed prior to MLA. An MRI should be collected and evaluated after any surgical procedure (MLA, surgical resection/debulking, or biopsy).

MRI should also be collected prior to first dose of MK-3475 for patients not receiving any surgical procedure.

Clinical lesions: Clinical lesions will only be considered measurable on brain MRI when they are ≥ 10 mm in bi-dimensional diameters as assessed using a ruler.

Histology: This technique can be used to differentiate between partial responses (PR) and complete responses (CR) in rare cases when biopsy or surgical resection of a measureable lesion is clinically indicated.

Perfusion/CBV: This advanced brain MRI technique can be used as an adjunct test to determine treatment response or disease status. However, it should not be used as the primary or sole method to determine response or disease status.

Brain FDG-PET coupled with head CT or brain MRI: This advanced metabolic imaging technique can be used as an adjunct test to determine response or disease status. However it should be used as the primary or sole method of determining response or disease status.

12.3.1 Evaluation of Target Lesions

Complete Response (CR): Disappearance of all target lesions.

Partial Response (PR): ≥ 50% decrease compared with baseline in the sum of products of perpendicular diameters of all target lesions sustained for at least 4 weeks.

Progressive Disease (PD): At least a 25% increase in the sum of products of perpendicular diameters of at least 1 target lesion, taking as reference the smallest sum of products of perpendicular diameters on study (this includes the baseline sum if that is the smallest on study). The absolute increase in any dimension must be at least 5mm when calculating the products of perpendicular diameters.

In the event of surgical intervention for clinical reasons of a radiographically progressing tumor when subsequent surgical pathology demonstrates a predominant immune-mediated reaction **with no obvious progression of tumor**, the subject will be deemed not progressed and allowed to resume study treatment as assigned prior to the surgery at a time deemed safe by the treating physician, but no later than 8 weeks from the surgery.

Stable Disease (SD): Neither sufficient shrinkage to qualify for PR nor sufficient increase to qualify for PD, taking as reference the smallest sum of products of perpendicular diameters while on study.

12.3.2 Evaluation of Non-Target Lesions

Complete Response (CR): Disappearance of all non-target lesions.

Non-CR/Non-PD: Persistence of one or more non-target lesion(s).

Progressive Disease (PD): Appearance of one or more new lesions and/or unequivocal progression of existing non-target lesions on stable or increasing doses of corticosteroids compared with baseline scan or best response after initiation of therapy* not caused by comorbid events (e.g. radiation therapy, demyelination, ischemic injury, infection, seizures, postoperative changes, or other treatment effects). Unequivocal progression should not normally trump target lesion status. It must be representative of overall disease status change, not a single lesion increase.

Although a clear progression of “non-target” lesions only is exceptional, the opinion of the treating physician should prevail in such circumstances, and the progression status should be confirmed at a later time by the review panel (or Principal Investigator).

12.3.3 Evaluation of Best Overall Response

The best overall response is the best response recorded from the start of the treatment until disease progression/recurrence (taking as reference for progressive disease the smallest measurements recorded since the treatment started). The patient's best response assignment will depend on the achievement of both measurement and confirmation criteria.

Summary of the RANO Response Criteria (Adapted from JCO 2010)

Criterion	CR	PR	SD	PD
T1 gadolinium enhancing disease	None	≥ 50% ↓	< 50% ↓ but < 25% ↑	≥ 25% ↑*
T2/FLAIR	Stable or ↓	Stable or ↓	Stable or ↓	↑*
New lesion	None	None	None	Present*
Corticosteroids	None	Stable or ↓	Stable or ↓	NA†
Clinical status	Stable or ↑	Stable or ↑	Stable or ↑	↓*
Requirement for response	All	All	All	Any*

Abbreviations: RANO, Response Assessment in Neuro-Oncology; CR, complete response; PR, partial response; SD, stable disease; PD, progressive disease; FLAIR, fluid-attenuated inversion recovery; NA, not applicable.
* Progression occurs when this criterion is present.

† Increase in corticosteroids alone will not be taken into account in determining progression in the absence of persistent clinical deterioration.

NOTE: Patients may continue on treatment and remain under close observation and evaluation at 4-8 week intervals if there is uncertainty regarding whether pseudoprogression may be present as determined by the investigators. If subsequent radiographic or clinical assessments suggest that the patient is in fact experiencing progression, then the date of progression should be the time point at which this issue was first raised. Similarly, stable disease may be assigned in cases of presumed "pseudoprogression" associated with decreased steroid use.

12.3.4 Duration of Response

Duration of overall response: The duration of overall response is measured from the time measurement criteria are met for CR or PR (whichever is first recorded) until the first date that recurrent or progressive disease is objectively documented (taking as reference for progressive disease the smallest measurements recorded since the treatment started).

The duration of overall CR is measured from the time measurement criteria are first met for CR until the first date that progressive disease is objectively documented.

Duration of stable disease: Stable disease is measured from the start of the treatment until the criteria for progression are met, taking as reference the smallest measurements recorded since the treatment started, including the baseline measurements.

12.3.5 Neurological Exam and Performance Status

Patients will be graded using the Karnofsky Performance Status scale and their neurological function evaluated as improved, stable or deteriorated in addition to objective measurement of tumor size. These parameters will be used to determine the overall response assessment.

12.3.6 Progression-Free Survival

PFS is defined as the duration of time from start of treatment to time of progression or death, whichever occurs first.

13.0 DATA AND SAFETY MONITORING

In compliance with the Washington University Institutional Data and Safety Monitoring Plan, an independent Data and Safety Monitoring Board (DSMB) will be specifically convened for this trial to review toxicity data. A DSMB will consist of no fewer than 3 members including 2 clinical investigators and a biostatistician. Like investigators, DSMB members are subject to the Washington University School of Medicine policies regarding standards of conduct. Individuals invited to serve on the DSMB will disclose any potential conflicts of interest to the trial principal investigator and/or appropriate university officials, in accordance with institution policies.

Potential conflicts that develop during a trial or a member's tenure on a DSMB must also be disclosed.

Until such a time as the first secondary site enrolls its first patient, a semi-annual DSM report to be prepared by the study team will be submitted to the Quality Assurance and Safety Monitoring Committee (QASMC) semi-annually beginning six months after study activation at Washington University (if at least one patient has been enrolled) or one year after study activation (if no patients have been enrolled at the six-month mark).

The DSM report will be prepared by the study team with assistance from the study statistician, will be reviewed by the DSMB, and will be submitted to the Quality Assurance and Safety Monitoring Committee (QASMC). The DSMB must meet at least every six months beginning six months after study activation at Washington University or beginning six months after enrollment of the first patient at a secondary site, no more than one month prior to the due date of the DSM report to QASMC.

During the phase I dose escalation, the Principal Investigator will review all patient data at least monthly (or before each dose escalation if occurring sooner than monthly), and provide a semi-annual report to the QASMC. During the phase II, the Principal Investigator/DSMB will review all patient data at least every six months, and provide a semi-annual report to the QASMC. This report will include:

- HRPO protocol number, protocol title, Principal Investigator name, data coordinator name, regulatory coordinator name, and statistician
- Date of initial HRPO approval, date of most recent consent HRPO approval/revision, date of HRPO expiration, date of most recent QA audit, study status, and phase of study
- History of study including summary of substantive amendments; summary of accrual suspensions including start/stop dates and reason; and summary of protocol exceptions, error, or breach of confidentiality including start/stop dates and reason
- Study-wide target accrual and study-wide actual accrual including numbers from participating sites
- Protocol activation date at each participating site
- Average rate of accrual observed in year 1, year 2, and subsequent years at each participating site
- Expected accrual end date, accrual by site, and accrual by cohort
- Objectives of protocol with supporting data and list the number of participants who have met each objective
- Measures of efficacy
- Early stopping rules with supporting data and list the number of participants who have met the early stopping rules
- Summary of toxicities at all participating sites separated by cohorts with the number of dose-limiting toxicities indicated
- Abstract submissions/publications
- Summary of any recent literature that may affect the safety or ethics of the study

Further DSMC responsibilities are described in the DSMC charter.

Until such a time as the first secondary site activates this protocol, a semi-annual Data and Safety Monitoring report to be prepared by the study team will be submitted to the QASM Committee beginning 6 months after study activation at Washington University.

The study principal investigator and coordinator will monitor for serious toxicities on an ongoing basis. Once the principal investigator or coordinator becomes aware of an adverse event, the AE will be reported to the HRPO and QASMC according to institutional guidelines (please refer to Section 7.0).

Refer to the Washington University Quality Assurance and Data Safety Monitoring Committee Policies and Procedures for full details on the responsibilities of the DSMC at https://siteman.wustl.edu/research/clinical-research-resources/protocol-office-prmcqasmc/.

14.0 AUDITING

As coordinating center of this trial, Washington University (via the Quality Assurance and Safety Monitoring Committee (QASMC) will monitor each participating site to ensure that all protocol requirements are being met; that applicable federal regulations are being followed; and that best practices for patient safety and data collection are being followed per protocol. Participating sites will be asked to send copies of all audit materials, including source documentation. The audit notification will be sent to the Washington University Research Patient Coordinator, who will obtain the audit materials from the participating institution.

Notification of an upcoming audit will be sent to the research team one month ahead of the audit. Once accrual numbers are confirmed, and approximately 30 days prior to the audit, a list of the cases selected for review (up to 10 for each site) will be sent to the research team. However, if during the audit the need arises to review cases not initially selected, the research team will be asked to provide the additional charts within two working days.

Items to be evaluated include:

- Subject screening and enrollment
- Reporting of adverse events
- Maintenance of HIPAA compliance
- Completeness of regulatory documentation
- Completeness of participant documentation
- Acquisition of informed consent
- IRB documentation
- Issues of protocol adherence

Additional details regarding the auditing policies and procedures can be found at https://siteman.wustl.edu/wp-content/uploads/2015/10/QASMC-Policies-and-Procedures-03.31.2015.pdf

15.0 STATISTICAL CONSIDERATIONS

15.1 Power Analysis

In the phase I portion of the study, a traditional 3+3 design is used to determine the maximal tolerated dose (MTD) of MK-3475 when combined with MLA for the treatment of recurrent malignant gliomas.

Power analysis for phase II is based on PFS. Using a 1-sided alpha of 0.15, with an accrual interval of 20 months and additional follow-up of 6 months (study period in Phase II is 26 months), and assuming a progression free survival probability at 6 month (6-PFS) of 40% for the MK-3475 alone group and 65% for MK-3475 + MLA combination group, we will need 17 patients per group with probability (power) 0.8. If the 6-PFS of the MK-3475 alone group is 45% and 65% for the MK-3475 + MLA combination group, we will need 25 patients per group. We are planning to enroll 20 patients per group for the phase II portion of trial.

6-PFS in MK-3475 alone group	6-PFS in MK-3475 + MLA combination group	Sample per group
40%	50%	108
	55%	48
	60%	27
	65%	17
45%	55%	105
	60%	46
	65%	25
	70%	16
50%	60%	100
	65%	43
	70%	24
	75%	15
55%	65%	93
	70%	40
	75%	21
	80%	13
60%	70%	84
	75%	35
	80%	19
	85%	11

Six-PFS for all current commonly used treatments for recurrent GBM (e.g. bevacizumab, temozolomide, carboplatin, carmustine, lomustine, etoposide, irinotecan) ranges from < 10% to low 30% [39-43]. Since historical data on 6-PFS in recurrent GBM for both MK-3475 and MLA are not available, for the purpose of reasonable estimation of efficacy of the MK-3475 and MLA combination as compared to MK-3475 alone in recurrent/progressive

GBM, we will assume that the 6-PFS of the MK-3475 alone group to be 40-45% to derive sample size estimates for both groups as detailed above.

15.2 Analysis Plan

PFS and OS will be estimated by Kaplan-Meier product limit method and the difference in PFS with recurrent GBM being treated with MK-3475 alone vs. those being treated with MK-3475 plus MLA will be compared by log-rank test.

Two-group t-test will be used to compare the anti-glioma immune response before and after MK-3475 with or without MLA.

Cox proportional hazard model will be used to determine the relationship between intratumoral expression of PD-L1, the frequency of glioma cell-specific cytotoxic T cells with PFS and OS while proportional hazard assumption will be examined.

Pearson correlation coefficient (r) will be calculated between intratumoral expression of PD-L1 and the frequency of glioma cell-specific cytotoxic T cells. Similarly, Cox proportional hazard model will be used to investigate the relationship between PD-1-dependent biomarkers in glioma cell-specific T cells and PFS and OS.

15.3 Phase II Stopping Rules

Toxicity will be reviewed on a continuous basis. Early stopping of this trial is calculated based upon the grade 3 seizure rate. It is assumed that a maximum-tolerated seizure rate is 50% and a seizure rate of 33% and or less is acceptable. For each treatment arm (n=20), based on the repeated significance testing (using R function toxbdry) with 80% power and 0.05 overall Type I error, the study will be halted if a treatment-related seizure occurs in the first patient, or both of the first 2 patients, or 3 of the first 4, or 4 of the first 5, or 5 of the first 7, or 6 of the first 9, or 7 of the first 11, or 8 of the first 14, or 9 of the first 16, or 10 of the first 18, or if the 11th treatment-related seizure is observed before the last (20th) patient has completed the trial.

In addition, enrollment will stop and a review will be conducted to determine whether modifications to the protocol are warranted or if the trial should be terminated if 2 patients in either group die or incur life-threatening (grade 4) adverse reactions that are thought to be at least possibly related to study treatment.

15.4 Interim Analysis

Per DSMC request, an interim analysis was conducted in June 2019. The MK-3475 + MLA group included 2 GBM patients who received 200mg in Phase 1 and 10 patients in Phase 2. PFS probability at 6, 9, and 12 months after treatment are 45.0%, 45.0%, and 33.8%, the median PFS is 5.49 months. Survival probabilities at 6, 9, and 12 months after treatment are 75.0%, 62.5%, and 50.0%, and the median OS is not estimable. For the MK-3475 monotherapy (no MLA) group, PFS probability at 3 months after treatment is 44.4%

and the median PFS is 2.73 months (95% CI: 1.41-3.88). Survival probabilities at 3 and 6 months after treatment are 83.3%, and 31.3%, and the median OS is 4.77 months (95% CI: 1.74-8.78).

This analysis showed significant differences in PFS and survival probabilities between the MK-3475 + MLA group and the MK-3475 alone group. Therefore, with Amendment 12, we are revising the study to a single arm (MK-3475 + MLA) in the spirit of minimizing harm, potential or perceived, to subjects and to complete the study in a timely manner. The recent study[46] shows median PFS was 2.4 months. To be conservative, we will use the shorter mPFS for MK-3475 + MLA group and longer mPFS from historical data. We will enroll additional 27 patients and expect 20 GBM patients will be evaluable for this study. With an accrual interval of 20 months and additional follow-up of 6 months, a two-sided, one-sample logrank test calculated from a sample of 32 subjects achieves 80.9% power at a 0.05 significance level to detect a median PFS of 5.1 months in the MK-3475 + MLA group when the median PFS of the historical control is 2.9 months.

15.5 Subgroup analysis

For participants in the Second Course Phase (Retreatment), PFS and OS will be estimated by Kaplan-Meier product limit method.

16.0 MULTICENTER REGULATORY REQUIREMENTS

Washington University requires that each participating site sends its informed consent document to be reviewed and approved by the Washington University Regulatory Coordinator (or designee) prior to IRB/IEC submission.

Site activation is defined as when the secondary site has received official written documentation from the coordinating center that the site has been approved to begin enrollment. At a minimum, each participating institution must have the following documents on file at Washington University prior to study activation:

- Documentation of IRB approval of the study in the form of a letter or other official document from the participating institution's IRB. This documentation must show which version of the protocol was approved by the IRB.
- Documentation of IRB approval of an informed consent form. The consent must include a statement that data will be shared with Washington University, including the Quality Assurance and Safety Monitoring Committee (QASMC), the DSMC (if applicable), and the Washington University study team.
- Documentation of FWA, signed FDA Form 1572 (if applicable), and the CVs of all participating investigators.
- Protocol signature page signed and dated by the investigator at each participating site.

The coordinating center Principal Investigator (or designee) is responsible for disseminating to the participating sites all study updates, amendments, reportable adverse events, etc. Protocol/consent modifications and IB updates will be forwarded electronically to the secondary sites within 4

weeks of obtaining Washington University IRB approval. Activated secondary sites are expected to submit protocol/consent/IB modifications to their local IRBs within 4 weeks of receipt unless otherwise noted. Upon the secondary sites obtaining local IRB approval, documentation of such shall be sent to the Washington University study team within 2 weeks of receipt of approval.

Documentation of participating sites' IRB approval of annual continuing reviews, protocol amendments or revisions, all SAE reports, and all protocol violations/deviations/exceptions must be kept on file at Washington University.

The investigator or a designee from each institution must participate in a regular conference call to update and inform regarding the progress of the trial.

17.0 REFERENCES

1. Parkin DM. Global cancer statistics in the year 2000. The lancet oncology 2001;2:533-43.
2. Stupp R, Mason WP, van den Bent MJ, et al. Radiotherapy plus concomitant and adjuvant temozolomide for glioblastoma. The New England journal of medicine 2005;352:987-96.
3. Stupp R, Hegi ME, Mason WP, et al. Effects of radiotherapy with concomitant and adjuvant temozolomide versus radiotherapy alone on survival in glioblastoma in a randomised phase III study: 5-year analysis of the EORTC-NCIC trial. The lancet oncology 2009;10:459-66.
4. Adamson C, Kanu OO, Mehta AI, et al. Glioblastoma multiforme: a review of where we have been and where we are going. Expert opinion on investigational drugs 2009;18:1061-83.
5. Hawasli AH, Ray WZ, Murphy RK, Dacey RG, Jr., Leuthardt EC. Magnetic resonance imaging-guided focused laser interstitial thermal therapy for subinsular metastatic adenocarcinoma: technical case report. Neurosurgery 2012;70:332-7; discussion 8.
6. Hawasli AH, Kim AH, Dunn GP, Tran DD, Leuthardt EC. Stereotactic laser ablation of high-grade gliomas. Neurosurgical focus 2014;37:E1.
7. Mohammadi AM, Hawasli AH, Rodriguez A, et al. The role of laser interstitial thermal therapy in enhancing progression-free survival of difficult-to-access high-grade gliomas: a multicenter study. Cancer medicine 2014;3:971-9.
8. Hawasli AH, Bagade S, Shimony JS, Miller-Thomas M, Leuthardt EC. Magnetic resonance imaging-guided focused laser interstitial thermal therapy for intracranial lesions: single-institution series. Neurosurgery 2013;73:1007-17.
9. Dix AR, Brooks WH, Roszman TL, Morford LA. Immune defects observed in patients with primary malignant brain tumors. Journal of neuroimmunology 1999;100:216-32.
10. Dunn GP, Dunn IF, Curry WT. Focus on TILs: Prognostic significance of tumor infiltrating lymphocytes in human glioma. Cancer immunity 2007;7:12.
11. Dunn GP, Fecci PE, Curry WT. Cancer immunoediting in malignant glioma. Neurosurgery 2012;71:201-22; discussion 22-3.
12. Fecci PE, Mitchell DA, Whitesides JF, et al. Increased regulatory T-cell fraction amidst a diminished CD4 compartment explains cellular immune defects in patients with malignant glioma. Cancer research 2006;66:3294-302.
13. Okada H, Kalinski P, Ueda R, et al. Induction of CD8+ T-cell responses against novel glioma-associated antigen peptides and clinical activity by vaccinations with {alpha}-type 1 polarized dendritic cells and polyinosinic-polycytidylic acid stabilized by lysine and carboxymethylcellulose in patients with recurrent malignant glioma. Journal of clinical oncology : official journal of the American Society of Clinical Oncology 2011;29:330-6.
14. Hamid O, Robert C, Daud A, et al. Safety and tumor responses with lambrolizumab (anti-PD-1) in melanoma. The New England journal of medicine 2013;369:134-44.
15. Lai A, Tran A, Nghiemphu PL, et al. Phase II study of bevacizumab plus temozolomide during and after radiation therapy for patients with newly diagnosed glioblastoma multiforme. Journal of clinical oncology : official journal of the American Society of Clinical Oncology 2011;29:142-8.
16. Disis ML. Immune regulation of cancer. Journal of clinical oncology : official journal of the American Society of Clinical Oncology 2010;28:4531-8.
17. Dong H, Strome SE, Salomao DR, et al. Tumor-associated B7-H1 promotes T-cell apoptosis: a potential mechanism of immune evasion. Nature medicine 2002;8:793-800.

18. Sharpe AH, Freeman GJ. The B7-CD28 superfamily. Nature reviews Immunology 2002;2:116-26.
19. Brown JA, Dorfman DM, Ma FR, et al. Blockade of programmed death-1 ligands on dendritic cells enhances T cell activation and cytokine production. Journal of immunology 2003;170:1257-66.
20. Francisco LM, Sage PT, Sharpe AH. The PD-1 pathway in tolerance and autoimmunity. Immunological reviews 2010;236:219-42.
21. Thompson RH, Dong H, Lohse CM, et al. PD-1 is expressed by tumor-infiltrating immune cells and is associated with poor outcome for patients with renal cell carcinoma. Clinical cancer research : an official journal of the American Association for Cancer Research 2007;13:1757-61.
22. Talmadge JE, Donkor M, Scholar E. Inflammatory cell infiltration of tumors: Jekyll or Hyde. Cancer metastasis reviews 2007;26:373-400.
23. Usubutun A, Ayhan A, Uygur MC, Ozen H, Toklu C, Ruacan S. Prognostic factors in renal cell carcinoma. Journal of experimental & clinical cancer research : CR 1998;17:77-81.
24. Al-Shibli KI, Donnem T, Al-Saad S, Persson M, Bremnes RM, Busund LT. Prognostic effect of epithelial and stromal lymphocyte infiltration in non-small cell lung cancer. Clinical cancer research : an official journal of the American Association for Cancer Research 2008;14:5220-7.
25. Deschoolmeester V, Baay M, Van Marck E, et al. Tumor infiltrating lymphocytes: an intriguing player in the survival of colorectal cancer patients. BMC immunology 2010;11:19.
26. Diez M, Pollan M, Enriquez JM, et al. Histopathologic prognostic score in colorectal adenocarcinomas. Anticancer research 1998;18:689-94.
27. Galon J, Costes A, Sanchez-Cabo F, et al. Type, density, and location of immune cells within human colorectal tumors predict clinical outcome. Science 2006;313:1960-4.
28. Hiraoka N. Tumor-infiltrating lymphocytes and hepatocellular carcinoma: molecular biology. International journal of clinical oncology 2010;15:544-51.
29. Nobili C, Degrate L, Caprotti R, et al. Prolonged survival of a patient affected by pancreatic adenocarcinoma with massive lymphocyte and dendritic cell infiltration after interleukin-2 immunotherapy. Report of a case. Tumori 2008;94:426-30.
30. Hodi FS, Dranoff G. The biologic importance of tumor-infiltrating lymphocytes. Journal of cutaneous pathology 2010;37 Suppl 1:48-53.
31. Kloor M. Lymphocyte infiltration and prognosis in colorectal cancer. The lancet oncology 2009;10:840-1.
32. Hillen F, Baeten CI, van de Winkel A, et al. Leukocyte infiltration and tumor cell plasticity are parameters of aggressiveness in primary cutaneous melanoma. Cancer immunology, immunotherapy : CII 2008;57:97-106.
33. Lee HE, Chae SW, Lee YJ, et al. Prognostic implications of type and density of tumour-infiltrating lymphocytes in gastric cancer. British journal of cancer 2008;99:1704-11.
34. Leffers N, Gooden MJ, de Jong RA, et al. Prognostic significance of tumor-infiltrating T-lymphocytes in primary and metastatic lesions of advanced stage ovarian cancer. Cancer immunology, immunotherapy : CII 2009;58:449-59.
35. Nishimura H, Honjo T, Minato N. Facilitation of beta selection and modification of positive selection in the thymus of PD-1-deficient mice. The Journal of experimental medicine 2000;191:891-8.

36. Liotta F, Gacci M, Frosali F, et al. Frequency of regulatory T cells in peripheral blood and in tumour-infiltrating lymphocytes correlates with poor prognosis in renal cell carcinoma. BJU international 2011;107:1500-6.
37. Flomenberg P, Piaskowski V, Truitt RL, Casper JT. Characterization of human proliferative T cell responses to adenovirus. J Infect Dis 1995;171:1090-6.
38. Tang J, Flomenberg P, Harshyne L, Kenyon L, Andrews DW. Glioblastoma patients exhibit circulating tumor-specific CD8+ T cells. Clin Cancer Res 2005;11:5292-9.
39. Franceschi E, Cavallo G, Scopece L, et al. Phase II trial of carboplatin and etoposide for patients with recurrent high-grade glioma. British journal of cancer 2004;91:1038-44.
40. Reardon DA, Quinn JA, Rich JN, et al. Phase I trial of irinotecan plus temozolomide in adults with recurrent malignant glioma. Cancer 2005;104:1478-86.
41. Brandes AA, Tosoni A, Amista P, et al. How effective is BCNU in recurrent glioblastoma in the modern era? A phase II trial. Neurology 2004;63:1281-4.
42. Kappelle AC, Postma TJ, Taphoorn MJ, et al. PCV chemotherapy for recurrent glioblastoma multiforme. Neurology 2001;56:118-20.
43. Chua SL, Rosenthal MA, Wong SS, et al. Phase 2 study of temozolomide and Caelyx in patients with recurrent glioblastoma multiforme. Neuro-oncology 2004;6:38-43.
44. Omuro A, Vlahovic G, Lim M, et al. Nivolumab with or without ipilimumab in patients with recurrent glioblastoma: results from exploratory phase I cohorts of CheckMate 143. Neuro-Oncology 2017;20:674-86.
45. Reardon DA, Nayak L, Peters KB, et al. Phase II study of pembrolizumab or pembrolizumab plus bevacizumab for recurrent glioblastoma (rGBM) patients. 2018;36:2006-2006.
46. Cloughesy TF, Mochizuki AY, Orpilla JR, et al. Neoadjuvant anti-PD-1 immunotherapy promotes a survival benefit with intratumoral and systemic immune responses in recurrent glioblastoma. Nature Medicine 2019;25:477-86.

APPENDIX A: Karnofsky Performance Scale

Able to carry on normal activity and to work; no special care needed.	100	Normal no complaints; no evidence of disease.
	90	Able to carry on normal activity; minor signs or symptoms of disease.
	80	Normal activity with effort; some signs or symptoms of disease.
Unable to work; able to live at home and care for most personal needs; varying amount of assistance needed.	70	Cares for self; unable to carry on normal activity or to do active work.
	60	Requires occasional assistance, but is able to care for most of his personal needs.
	50	Requires considerable assistance and frequent medical care.
Unable to care for self; requires equivalent of institutional or hospital care; disease may be progressing rapidly.	40	Disabled; requires special care and assistance.
	30	Severely disabled; hospital admission is indicated although death not imminent.
	20	Very sick; hospital admission necessary; active supportive treatment necessary.
	10	Moribund; fatal processes progressing rapidly.
	0	Dead

APPENDIX B: Definitions for Adverse Event Reporting

A. Adverse Events (AEs)

As defined in 21 CFR 312.32:

Definition: any untoward medical occurrence associated with the use of a drug in humans, whether or not considered drug-related.

Grading: the descriptions and grading scales found in the revised NCI Common Terminology Criteria for Adverse Events (CTCAE) version 4.0 will be utilized for all toxicity reporting. A copy of the CTCAE version 4.0 can be downloaded from the CTEP website.

Attribution (relatedness), Expectedness, and Seriousness: the definitions for the terms listed that should be used are those provided by the Department of Health and Human Services' Office for Human Research Protections (OHRP). A copy of this guidance can be found on OHRP's website:
http://www.hhs.gov/ohrp/policy/advevntguid.html

B. Suspected Adverse Reaction (SAR)

As defined in 21 CFR 312.32:

Definition: any adverse event for which there is a reasonable possibility that the drug caused the adverse event. "Reasonable possibility" means there is evidence to suggest a causal relationship between the drug and the adverse event. "Suspected adverse reaction" implies a lesser degree of certainty about causality than adverse reaction, which means any adverse event caused by a drug.

C. Life-Threatening Adverse Event / Life Threatening Suspected Adverse Reaction

As defined in 21 CFR 312.32:

Definition: any adverse drug event or suspected adverse reaction is considered "life-threatening" if, in the view of the investigator, its occurrence places the patient at immediate risk of death. It does not include an adverse event or suspected adverse reaction that, had it occurred in a more severe form, might have caused death.

D. Serious Adverse Event (SAE) or Serious Suspected Adverse Reaction

As defined in 21 CFR 312.32:

Definition: an adverse event or suspected adverse reaction is considered "serious" if, in the view of the investigator, it results in any of the following outcomes:

- Death
- A life-threatening adverse event
- Inpatient hospitalization or prolongation of existing hospitalization

- A persistent or significant incapacity or substantial disruption of the ability to conduct normal life functions
- A congenital anomaly/birth defect
- Any other important medical event that does not fit the criteria above but, based upon appropriate medical judgment, may jeopardize the subject and may require medical or surgical intervention to prevent one of the outcomes listed above

E. Events of Clinical Interest

Events of clinical interest for this trial include:

1. an overdose of Merck product that is not associated with clinical symptoms or abnormal laboratory results.
2. an elevated AST or ALT lab value that is ≥ 3 x IULN and an elevated total bilirubin lab value that is ≥ 2 x IULN and, at the same time, an alkaline phosphatase lab value that is < 2 x IULN, as determined by way of protocol-specified laboratory testing or unscheduled laboratory testing.

F. Protocol Exceptions

Definition: A planned change in the conduct of the research for one participant.

G. Deviation

Definition: Any alteration or modification to the IRB-approved research without prospective IRB approval. The term "research" encompasses all IRB-approved materials and documents including the detailed protocol, IRB application, consent form, recruitment materials, questionnaires/data collection forms, and any other information relating to the research study.

A minor or administrative deviation is one that does not have the potential to negatively impact the rights, safety, or welfare of participants or others or the scientific validity of the study.

A major deviation is one that does have the potential to negatively impact the rights, safety, or welfare of participants or others or the scientific validity of the study.

APPENDIX C: Reporting Timelines

Expedited Reporting Timelines				
**Event**	**HRPO**	**QASMC**	**FDA**	**Merck & Co.**
Serious AND unexpected suspected adverse reaction			Report no later than 15 calendar days after it is determined that the information qualifies for reporting	Report to Merck & Co. within 24 hours of Sponsor-Investigator notification.
Unexpected fatal or life-threatening suspected adverse reaction			Report no later than 7 calendar days after initial receipt of the information	
Unanticipated problem involving risk to participants or others	Report within 10 working days. If the event results in the death of a participant enrolled at WU/BJH/SLCH, report within 1 working day.	Report via email after IRB acknowledgment		
Pregnancy				Report to Merck & Co. within 24 hours of Sponsor-Investigator notification.
Overdose				Report to Merck & Co. within 24 hours of Sponsor-Investigator notification.
Events of Clinical Interest				Report to Merck & Co. within 24 hours of Sponsor-Investigator notification.
Major deviation	Report within 10 working days. If the event results in the death of a participant enrolled at WU/BJH/SLCH, report within 1 working day.			
A series of minor deviations that are being reported as a continuing noncompliance	Report within 10 working days.			
Protocol exception	Approval must be obtained prior to implementing the change			

Expedited Reporting Timelines				
**Event**	**HRPO**	**QASMC**	**FDA**	**Merck & Co.**
Clinically important increase in the rate of a serious suspected adverse reaction of that list in the protocol or IB			Report no later than 15 calendar days after it is determined that the information qualifies for reporting	
Complaints	If the complaint reveals an unanticipated problem involving risks to participants or others OR noncompliance, report within 10 working days. If the event results in the death of a participant enrolled at WU/BJH/SLCH, report within 1 working day. Otherwise, report at the time of continuing review.			
Breach of confidentiality	Within 10 working days.			
Incarceration	If withdrawing the participant poses a safety issue, report within 10 working days.<br><br>If withdrawing the participant does not represent a safety issue and the patient will be withdrawn, report at continuing review.			

Routine Reporting Timelines				
**Event**	**HRPO**	**QASMC**	**FDA**	**Merck & Co.**
Adverse event or SAE that does not require expedited reporting	If they do not meet the definition of an unanticipated problem involving risks to participants or others, report summary information at the time of continuing review	Adverse events will be reported in the toxicity table in the DSM report which is typically due every 6 months.	The most current toxicity table from the DSM report is provided to the FDA with the IND's annual report.	Notify monthly via AE notification or cross-reporting reports/documents.

Routine Reporting Timelines				
**Event**	**HRPO**	**QASMC**	**FDA**	**Merck & Co.**
Minor deviation	Report summary information at the time of continuing review.			
Complaints	If the complaint reveals an unanticipated problem involving risks to participants or others OR noncompliance, report within 10 working days. If the event results in the death of a participant enrolled at WU/BJH/SLCH, report within 1 working day. Otherwise, report at the time of continuing review.			
Incarceration	If withdrawing the participant poses a safety issue, report within 10 working days.<br><br>If withdrawing the participant does not represent a safety issue and the patient will be withdrawn, report at continuing review.			

Expedited Reporting Timelines for Secondary Sites				
**Event**	**WU (Coordinating Center)**	**Local IRB**	**FDA**	**Merck & Co.**
Serious AND unexpected suspected adverse reaction	Report no later than 11 calendar days after it is determined that the information qualifies for reporting.	Report all applicable events to local IRB according to local institutional guidelines.	The research team at Washington University is responsible for reporting all applicable events to the FDA as needed.	The research team at Washington University is responsible for reporting all applicable events to Polaris as needed.
Unexpected fatal or life-threatening suspected adverse reaction	Report no later than 4 calendar days after initial receipt of the information.			
Unanticipated problem involving risk to participants or others	Report no later than 4 calendar days after initial receipt of the information.			
Adverse event or SAE that does not require expedited reporting	As per routine data entry expectations			
Protocol exception	Approval must be obtained prior to implementing the change.			

APPENDIX D: Washington University Serious Adverse Event Reporting Cover Sheet

SAE COVER SHEET- Secondary Site Assessment

Washington University HRPO#:	Sponsor-Investigator:
Subject Initials:	Subject ID:
Treating MD:	Treating Site:
EVENT TERM:	Event Start Date:
EVENT GRADE:	Date of site's first notification:

Treating MD Event Assessment:

Is this event **possibly, probably, or definitely** related study treatment?

☐ yes ☐ no

If yes, please list which drug (if more than one)______________________________

Explain ____

____

_________________________ _________________________ _________________

Physician's Name **Physician's Signature** **Date**
