# Supplementary material for: Laser interstitial thermal therapy and adjuvant pembrolizumab in recurrent high-grade astrocytoma: a Phase 1/randomized Phase 2b trial
Source: Nat Commun. 2026 Feb 26;17:1763. doi: 10.1038/s41467-026-69522-w (PMC12946167; doi:10.1038/s41467-026-69522-w)
Supplement: Supplementary file 2 — Reporting Summary [file 41467_2026_69522_MOESM2_ESM.pdf]

nature portfolio

Corresponding author(s): David D. Tran

Last updated by author(s): 01/30/2026

Reporting Summary

Nature Portfolio wishes to improve the reproducibility of the work that we publish. This form provides structure for consistency and transparency in reporting. For further information on Nature Portfolio policies, see our Editorial Policies and the Editorial Policy Checklist.

Statistics

For all statistical analyses, confirm that the following items are present in the figure legend, table legend, main text, or Methods section.

n/a	Confirmed	
☐	☒	The exact sample size (*n*) for each experimental group/condition, given as a discrete number and unit of measurement
☐	☒	A statement on whether measurements were taken from distinct samples or whether the same sample was measured repeatedly
☐	☒	The statistical test(s) used AND whether they are one- or two-sided<br>*Only common tests should be described solely by name; describe more complex techniques in the Methods section.*
☐	☒	A description of all covariates tested
☐	☒	A description of any assumptions or corrections, such as tests of normality and adjustment for multiple comparisons
☐	☒	A full description of the statistical parameters including central tendency (e.g. means) or other basic estimates (e.g. regression coefficient) AND variation (e.g. standard deviation) or associated estimates of uncertainty (e.g. confidence intervals)
☐	☒	For null hypothesis testing, the test statistic (e.g. *F*, *t*, *r*) with confidence intervals, effect sizes, degrees of freedom and *P* value noted<br>*Give P values as exact values whenever suitable.*
☒	☐	For Bayesian analysis, information on the choice of priors and Markov chain Monte Carlo settings
☒	☐	For hierarchical and complex designs, identification of the appropriate level for tests and full reporting of outcomes
☒	☐	Estimates of effect sizes (e.g. Cohen's *d*, Pearson's *r*), indicating how they were calculated

Our web collection on statistics for biologists contains articles on many of the points above.

Software and code

Policy information about availability of computer code

Data collection	Data collection is described in detail in the Study Design section of the Methods
Data analysis	Data analysis is described in detail in the Study Design section of the Methods. Access information is provided in the Data and Code Availability section of the Methods and be found at: https://github.com/TranLabUSC/MK_clinical_trial_paper (https://doi.org/10.5281/zenodo.18330074)

For manuscripts utilizing custom algorithms or software that are central to the research but not yet described in published literature, software must be made available to editors and reviewers. We strongly encourage code deposition in a community repository (e.g. GitHub). See the Nature Portfolio guidelines for submitting code & software for further information.

Data

Policy information about availability of data

All manuscripts must include a data availability statement. This statement should provide the following information, where applicable:

- Accession codes, unique identifiers, or web links for publicly available datasets
- A description of any restrictions on data availability
- For clinical datasets or third party data, please ensure that the statement adheres to our policy

Data availability statement

The WES, bulk RNA-seq, scRNA-seq, and single-cell TCR sequencing data and associated clinical metadata generated in this study has been deposited in the European Genome-phenome Archive (EGA) under Study ID–EGAS50000001144 and Dataset ID–EGAD50000001639 (https://ega-archive.org/datasets/

EGAD50000001639). Access to these human genomics data is controlled to protect participant privacy and to ensure use consistent with informed consent and applicable ethics approvals. Access can be obtained by submitting a request through EGA; requests are reviewed and access is granted upon approval by the study's lead contact (david.tran@med.usc.edu) and completion of the EGA Data Access Agreement. Additional individual de-identified participant data will be shared upon reasonable request. Source data are provided with this paper. The remaining data are available within the Article, Supplementary Information or Source.

Code availability statement
All bioinformatics codes with source data are described in the Methods section and can be accessed at https://github.com/TranLabUSC/MK_clinical_trial_paper (https://doi.org/10.5281/zenodo.18330074)

Research involving human participants, their data, or biological material

Policy information about studies with human participants or human data. See also policy information about sex, gender (identity/presentation), and sexual orientation and race, ethnicity and racism.

Reporting on sex and gender	The study prospectively enrolled both male and female patients. In glioblastoma, men typically account for about 55% of cases versus 45% women, and our cohort reflected this distribution with 51.5% male participants. Sex was recorded as documented in the electronic medical record and included as a covariate in our Cox proportional hazards models. Although prior studies have demonstrated sex-specific differences in GBM prognosis and treatment response, our sample size precluded a meaningful investigation of these effects. Gender identity was not systematically collected.
Reporting on race, ethnicity, or other socially relevant groupings	Race and ethnicity were self-reported in the electronic medical record and extracted for this study. Although prior research has identified modest differences in GBM survival and treatment response by race and ethnicity, our limited sample size lacked sufficient power to examine these effects, so these variables were not included in our analyses.
Population characteristics	Key baseline characteristics for patients with recurrent high-grade glioma included age, sex, tumor location, IDH mutation status, MGMT promoter methylation status, KPS, number of prior recurrences, and treatment center.
Recruitment	Patients were enrolled on a rolling basis from our surgical, radiation oncology, and neuro-oncology clinics, as well as through referrals to our centers for treatment until the pre-specified number of evaluable patients was reached. To minimize selection bias, all rHGA patients were screened and enrolled if eligible. Data and tissue samples were collected by study coordinators in the clinic and outpatient infusion centers where these patients received treatment. All data and sample collections were performed according to the scheduled stipulated in the protocol and detailed in the Methods and Results sections
Ethics oversight	The study protocol was approved by the respective IRB at Washington University in St Louis, the University of Florida, and the University of Southern California. A written informed consent was obtained from each study participant before any study-related procedure was performed.

Note that full information on the approval of the study protocol must also be provided in the manuscript.

Field-specific reporting

Please select the one below that is the best fit for your research. If you are not sure, read the appropriate sections before making your selection.

☒ Life sciences ☐ Behavioural & social sciences ☐ Ecological, evolutionary & environmental sciences

For a reference copy of the document with all sections, see nature.com/documents/nr-reporting-summary-flat.pdf

Life sciences study design

All studies must disclose on these points even when the disclosure is negative.

Sample size	Phase 1: Nine patients were enrolled in a standard 3 + 3 dose-escalation design, with three patients assigned to each pembrolizumab dose level (100 mg, 150 mg, and 200 mg). All nine patients were evaluable for dose-limiting toxicities (DLT) to establish the recommended Phase 2 dose (RP2D).<br><br>Phase 2: Initial calculations for Phase 2b (one-sided α = 0.15) assumed a 6-month PFS rate of 40% in the NLS+PEM arm—based on published salvage-therapy PFS rates of 10–30%—and 65% in the LITT+PEM arm. This required 17 patients per arm to achieve 80% power, so we targeted 20 patients per arm. After 21 patients were randomized, emerging data showing limited ICI monotherapy benefit prompted an interim DSMC review, confirming significant PFS and OS advantages for LITT+PEM. Under this amendment, the control arm was closed, and the trial continued as a single-arm LITT+PEM cohort. Assuming a conservative 2.9-month median PFS for the NLS+PEM arm (the upper 95% confidence bound rather than the 2.4-month median as reported in the literature), enrolling 27 additional patients (to reach 20 evaluable GBM cases) provided 80.9% power (two-sided α = 0.05, log-rank) to detect an increase in median PFS to 5.1 months. In total, Phase 2 enrolled 45 patients: 21 in the randomized portion and 24 in the single-arm expansion.
Data exclusions	Phase 1: All nine enrolled patients were evaluable for dose-limiting toxicity and included in the DLT analysis.<br><br>Phase 2: A total of 45 patients were enrolled—21 in the randomized ITT cohort and 24 in the single-arm expansion. In the ITT cohort, two patients randomized to NLS+PEM withdrew consent to receive off-trial LITT+PEM, and three patients randomized to LITT+PEM without confirmed grade 4 histology were treated off-trial with chemotherapy. Of the 24 post-amendment enrollees, one was excluded due to insurance denial for LITT and three for unconfirmed grade 4 disease (two of whom were subsequently lost to follow-up). Ultimately, 30 Phase

	2 patients received LITT+PEM (10 randomized, 20 post-amendment). For the per-protocol analysis, we added three Phase 1 patients treated at the RP2D (dose level 3) who met Phase 2 eligibility, yielding 33 evaluable LITT+PEM and six evaluable NLS+PEM patients (n = 39; see Figure 1a).<br><br>For whole-exome sequencing, we used all available tumor tissue. Because LITT+PEM patients undergo only a biopsy at the time of ablation for diagnostic confirmation, residual tissue for research was often limited.
Replication	For analyses of survival, response, and toxicity, we incorporated all available clinical data. Similarly, our bioinformatics pipeline leveraged the full set of sequencing and clinical variables—including all Cox proportional hazards models.<br><br>For visualization of single-cell clusters (e.g., non-classical monocyte density maps and UMAP gene-expression distributions before and after LITT for the 500-gene signature in Figure 4, CD8$^+$ T-cell overlay density contours and optimal transport transitions in Figure 6, and central memory CD8$^+$ T-cell proliferative capacity in Figure 7b), we randomly sampled an equal number of cells at each time point to ensure consistent visual comparisons. We repeated this sampling procedure at least three times, observing the same qualitative patterns.<br><br>All quantitative cell-count and pathway-activation metrics, however, were computed using the complete set of cells within each cluster or subtype, so they are not influenced by the sampling used for visualization. For cluster enumeration—such as the CD8$^+$ T-cell subtype proportions shown in Figure 7a—we report the ratio of cells in a given cluster relative to the total T-cell population, which likewise remains unaffected by the visualization sampling.
Randomization	The study originally specified 1:1 randomization between LITT+PEM and NLS+PEM, which proceeded for the first 21 patients. When emerging data demonstrated limited benefit from NLS+PEM, a subsequent amendment then redirected all remaining enrollments (n = 24) into the LITT +PEM arm.
Blinding	The study was not blinded, as it evaluated the immunomodulatory effects of two fundamentally different surgical approaches (LITT versus non-LITT conventional surgery), rendering participant and investigator masking impractical.

Reporting for specific materials, systems and methods

We require information from authors about some types of materials, experimental systems and methods used in many studies. Here, indicate whether each material, system or method listed is relevant to your study. If you are not sure if a list item applies to your research, read the appropriate section before selecting a response.

Materials & experimental systems

n/a	Involved in the study
☒	☐ Antibodies
☒	☐ Eukaryotic cell lines
☒	☐ Palaeontology and archaeology
☒	☐ Animals and other organisms
☐	☒ Clinical data
☒	☐ Dual use research of concern
☒	☐ Plants

Methods

n/a	Involved in the study
☒	☐ ChIP-seq
☒	☐ Flow cytometry
☒	☐ MRI-based neuroimaging

Clinical data

Policy information about clinical studies

All manuscripts should comply with the ICMJE guidelines for publication of clinical research and a completed CONSORT checklist must be included with all submissions.

Clinical trial registration	NCT02311582
Study protocol	Attached
Data collection	Clinical data were captured prospectively per the approved protocol from clinic visits, hospital admissions, and medical records. Disease status was assessed by routine brain MRI using RANO criteria and, when indicated, by pathologic confirmation. Tumor specimens were obtained either at surgery or from archived tissue banks, and blood was drawn at the time of treatment visits. All tissue samples were de-identified before processing: RNA-seq library preparation was performed in our research laboratory, and sequencing was carried out by either our institutional core facility or an external accredited vendor.
Outcomes	Primary endpoint was PFS. Secondary endpoints were OS, ORR, safety, and immune signature. All survival endpoints were defined as the time from study enrollment to the survival events at the data cutoff date.

Plants

Seed stocks	N/A
Novel plant genotypes	N/A
Authentication	N/A
